# Supplementary material for: Unveiling structure-performance relationships from multi-scales in non-fullerene organic photovoltaics
Source: Nat Commun. 2021 Jul 30;12:4627. doi: 10.1038/s41467-021-24937-5 (PMC8324909; doi:10.1038/s41467-021-24937-5)
Supplement: Supplementary file 1 — Supplementary Information [file 41467_2021_24937_MOESM1_ESM.pdf]

Supplementary Information

Unveiling structure-performance relationships from multi-scales in non-fullerene organic photovoltaics

Shuixing Li[1,5], Lingling Zhan[1,5], Nannan Yao[2], Xinxin Xia[3], Zeng Chen[4], Weitao Yang[1], Chengliang He[1], Lijian Zuo[1,*], Minmin Shi,[1] Haiming Zhu,[4] Xinhui Lu,[3] Fengling Zhang[2] & Hongzheng Chen[1,*]

[1]State Key Laboratory of Silicon Materials, MOE Key Laboratory of Macromolecular Synthesis and Functionalization, Department of Polymer Science and Engineering, Zhejiang University, Hangzhou 310027, P. R. China.
[2]Department of Physics, Chemistry and Biology (IFM), Linköping University, Linköping 581 83, Sweden.
[3]Department of Physics, Chinese University of Hong Kong, New Territories, Hong Kong 999077, P. R. China.
[4]Department of Chemistry, Zhejiang University, Hangzhou 310027, P. R. China.
[5]These authors contributed equally: Shuixing Li, Lingling Zhan.
*email: zjuzlj@zju.edu.cn; hzchen@zju.edu.cn

Supplementary Figures

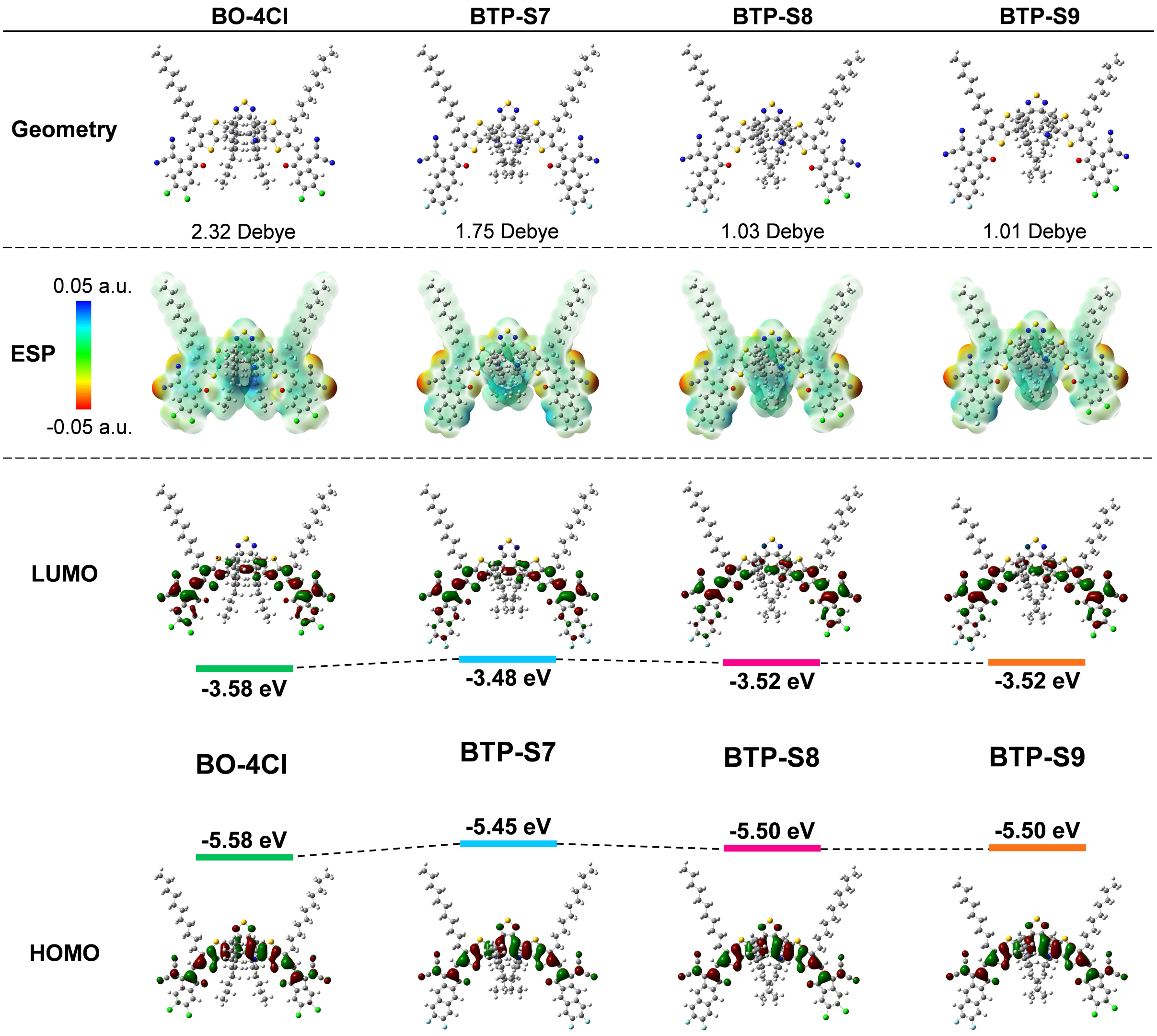

Supplementary Fig. 1 | Calculated geometries, electrostatic potential (ESP) and energy levels of BO-4Cl, BTP-S7, BTP-S8 and BTP-S9.

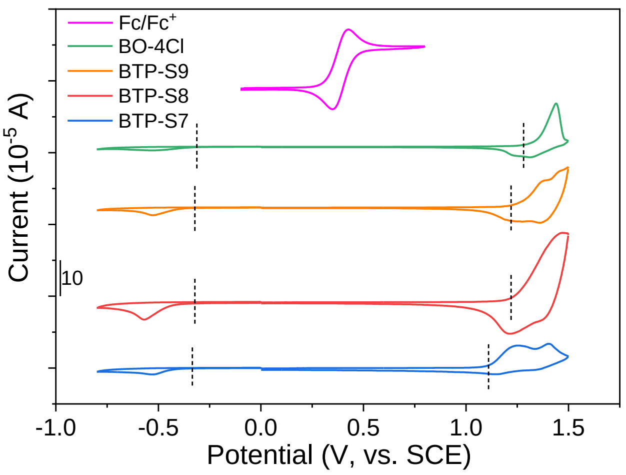

Supplementary Fig. 2 | Cyclic voltammograms of BO-4Cl, BTP-S7, BTP-S8, BTP-S9 and Fc/Fc^{+}.

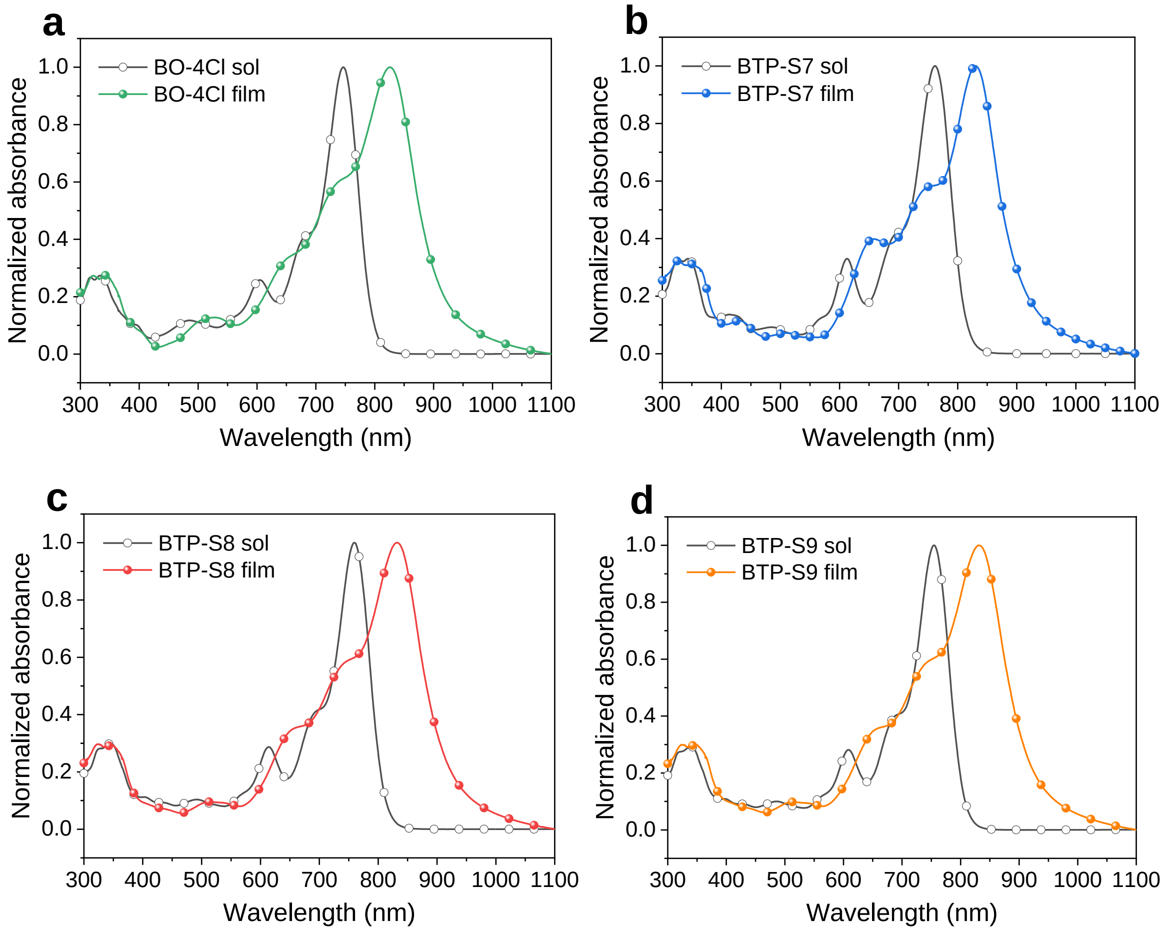

Supplementary Fig. 3 | Normalized absorption spectra of (a) BO-4Cl, (b) BTP-S7, (c) BTP-S8, (d) BTP-S9 chloroform solutions and thin films.

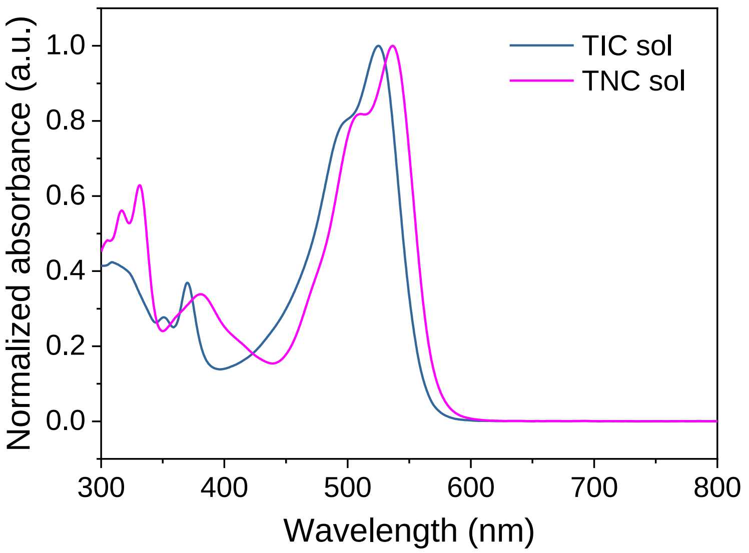

Supplementary Fig. 4 | Normalized absorption spectra of TIC and TNC in chloroform solutions.

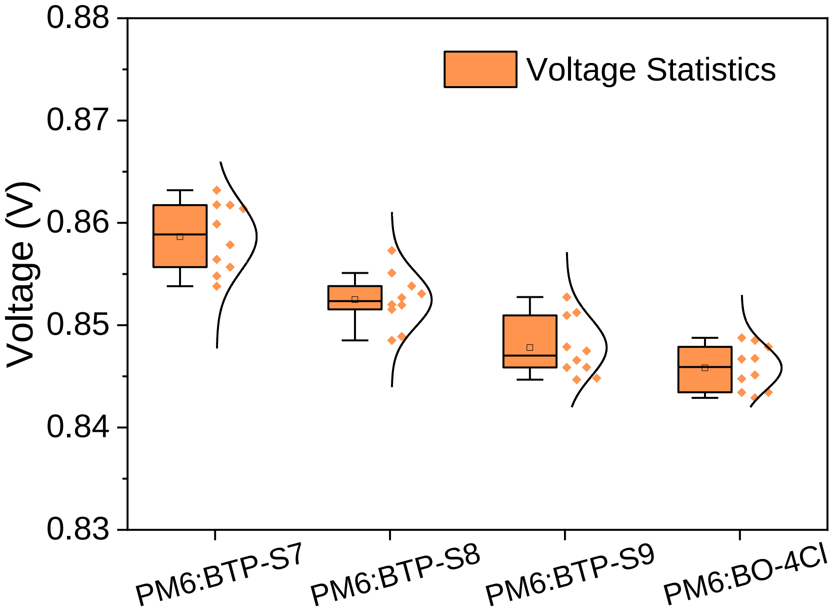

Supplementary Fig. 5 | Voltage statistics of OPVs based on PM6:BTP-S7, PM6:BTP-S8, PM6:BTP-S9 and PM6:BO-4Cl blends (error bar is defined as the standard deviation, which is calculated from the statistics results of ten devices).

Appendix: Summary of the Report
Report No.: GXgf2021-11242
Client:Zhejiang University
Sample: Organic Solar Cells
Type/Model: Organic Solar Cells
DUT S/N: S 1#-M1-F-1
Manufacturer: Zhejiang University
Date of Test: 05/13/2021
Temperature Sensor/Control System: None
Environmental conditions: (23.9±1) C°, RH (48.1±2) %
Mask: An aperture area of 4.728 mm^2 (M1, Certificate No.: CDjc2021-12227)

The test has been conducted by the PV Metrology Lab of NIM (National Institute of Metrology, China). Measurement of irradiance intensity and all other measurements are traceable to the International System of Units (SI). Data in this report apply only at the time of the test for the sample. For more details, please refer to the text of the report.

Forward Scan：

Area (mm^2)	I_{sc}(mA)	V_{oc}(V)	P_{max}(mW)
4.728	1.269	0.839	0.821
I_{max}(mA)	V_{max}(V)	FF (%)	η (%)
1.156	0.710	77.1	17.4

I-V Characterization Methods:
JJF 1622-2017: Calibration Specification of Solar Cells：Photoelectric Properties
Secondary Reference Cell:
Device S/N: 81#; Device Material: Mono-Si
Solar Simulator:
Classification: AAA (Double-light source: Xeon and Halogen);
Total irradiance: 1000 W/m^2 based on I_{sc} of the above Secondary Reference Cell.

Supplementary Fig. 6 | Efficiency certification report for OPV based on PM6:BTP-S9 blend from National Institute of Metrology (NIM), China.

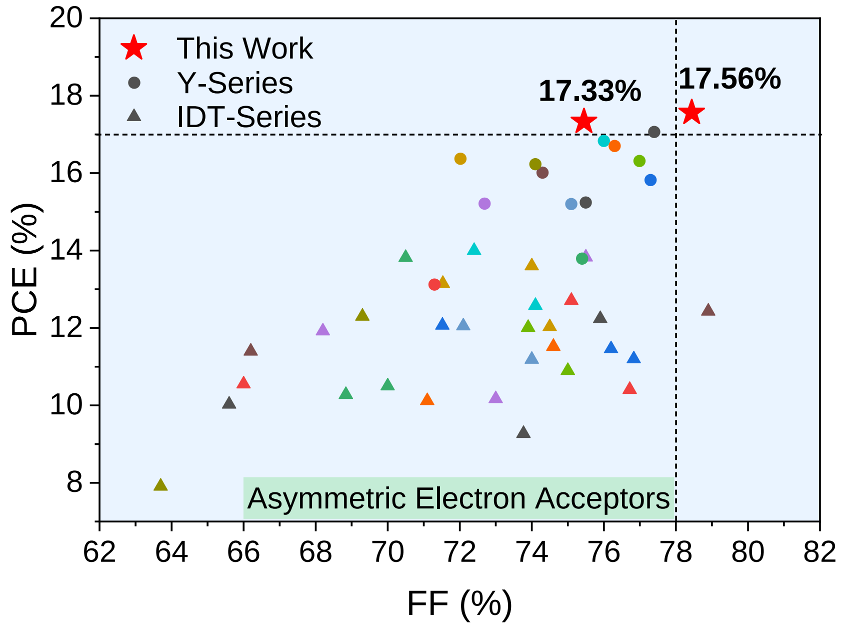

Supplementary Fig. 7 | Comparison of FF and PCE values between this work and reported references, raw data are summarized in **Supplementary Table 4**.

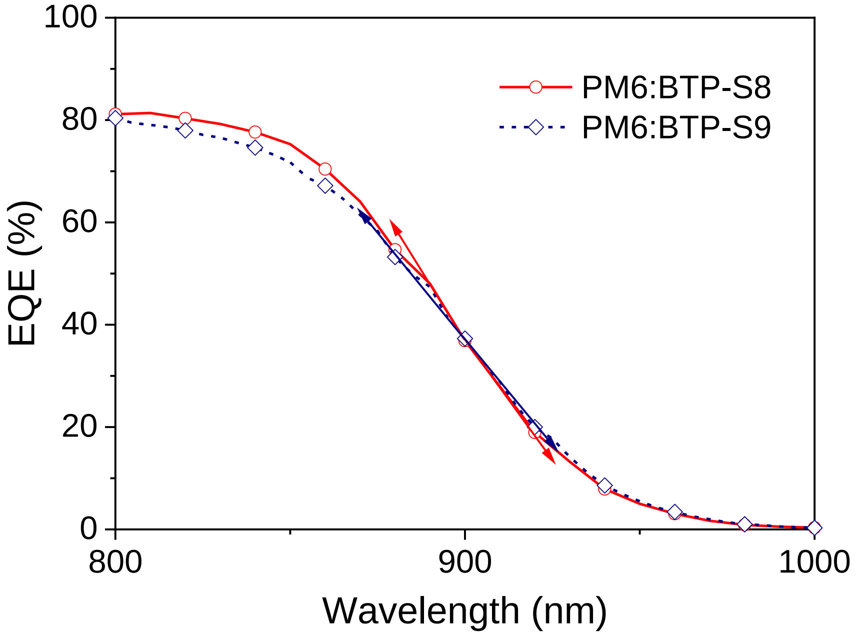

Supplementary Fig. 8 | Comparison of EQE curves of PM6:BTP-S8 and PM6:BTP-S9-based devices at the range of 800-1000 nm.

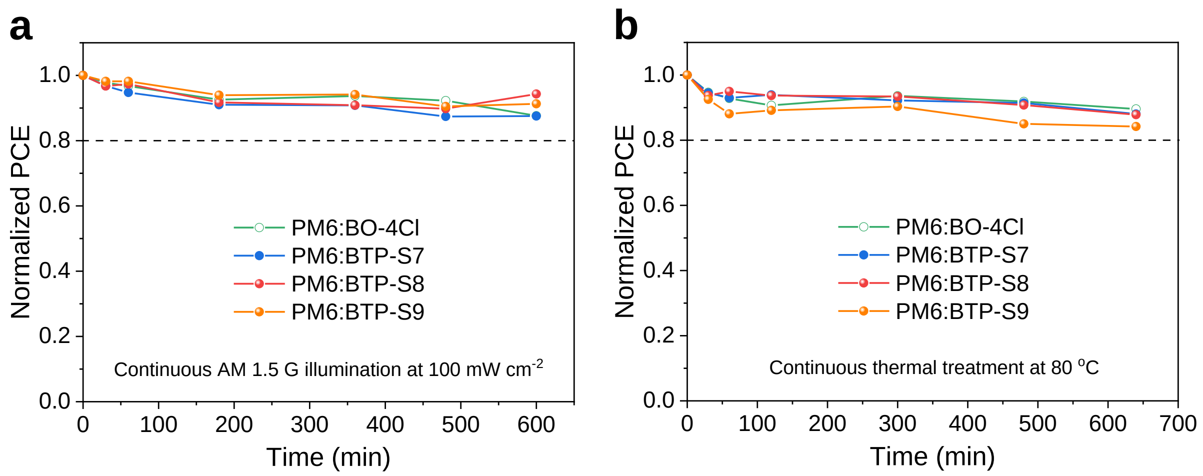

Supplementary Fig. 9 | a Light stability of four types of OPVs under continuous AM 1.5 G illumination at 100 mW cm^{-2} for various times. **b** Thermal stability of four types of OPVs under continuous thermal treatment at 80 °C for various times.

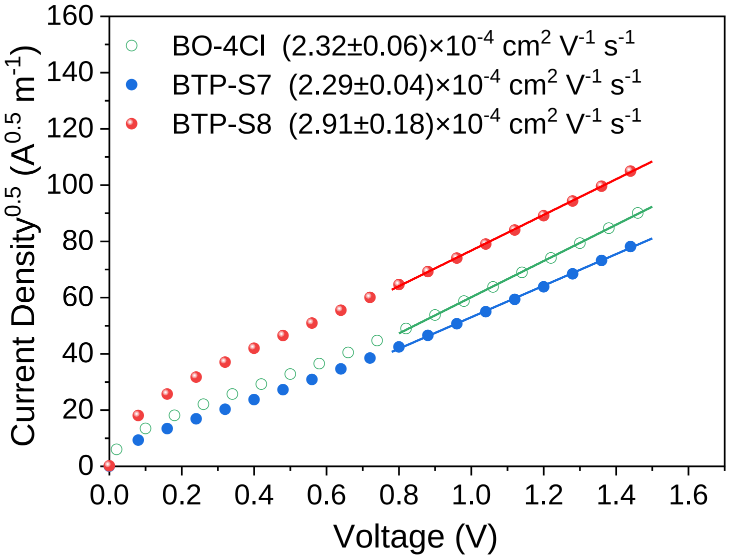

Supplementary Fig. 10 | $J^{0.5}$-V curves of the electron-only devices based on BO-4Cl, BTP-S7 and BTP-S8 neat films.

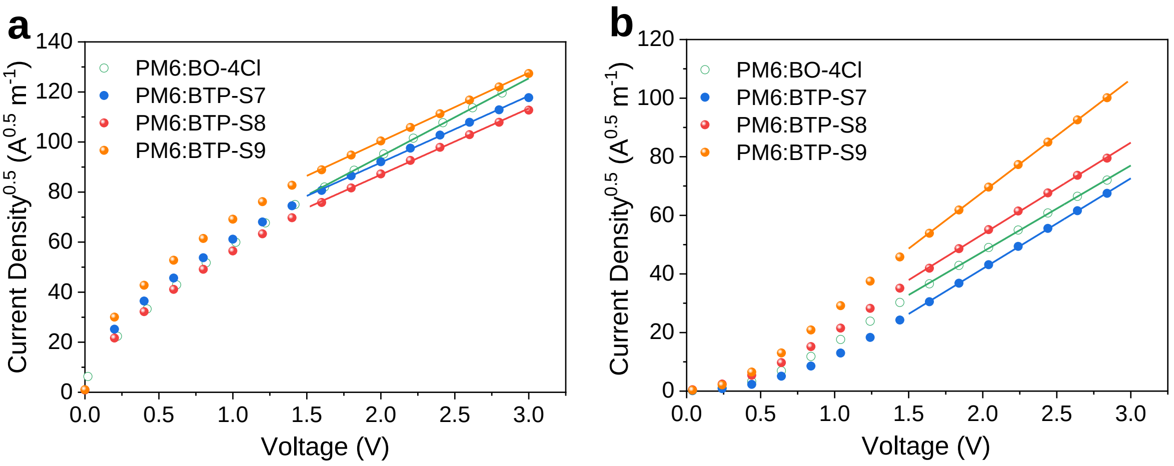

Supplementary Fig. 11 | a $J^{0.5}$-V curves of the hole-only devices based on PM6:BO-4Cl, PM6:BTP-S7, PM6:BTP-S8 and PM6:BTP-S9 blends. **b** $J^{0.5}$-V curves of the electron-only devices based on PM6:BO-4Cl, PM6:BTP-S7, PM6:BTP-S8 and PM6:BTP-S9 blends.

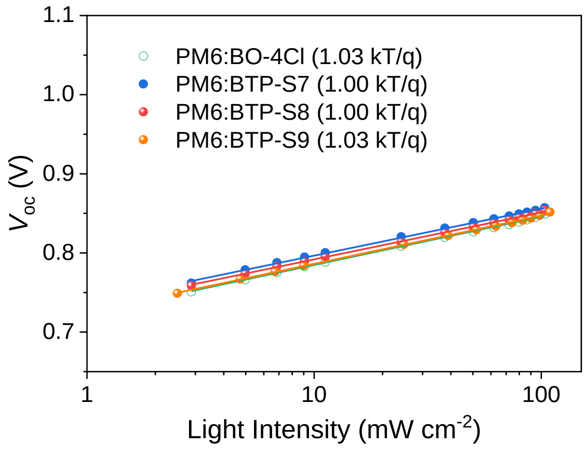

Supplementary Fig. 12 | The dependence of V_{oc} on P_{light} of relevant OPVs.

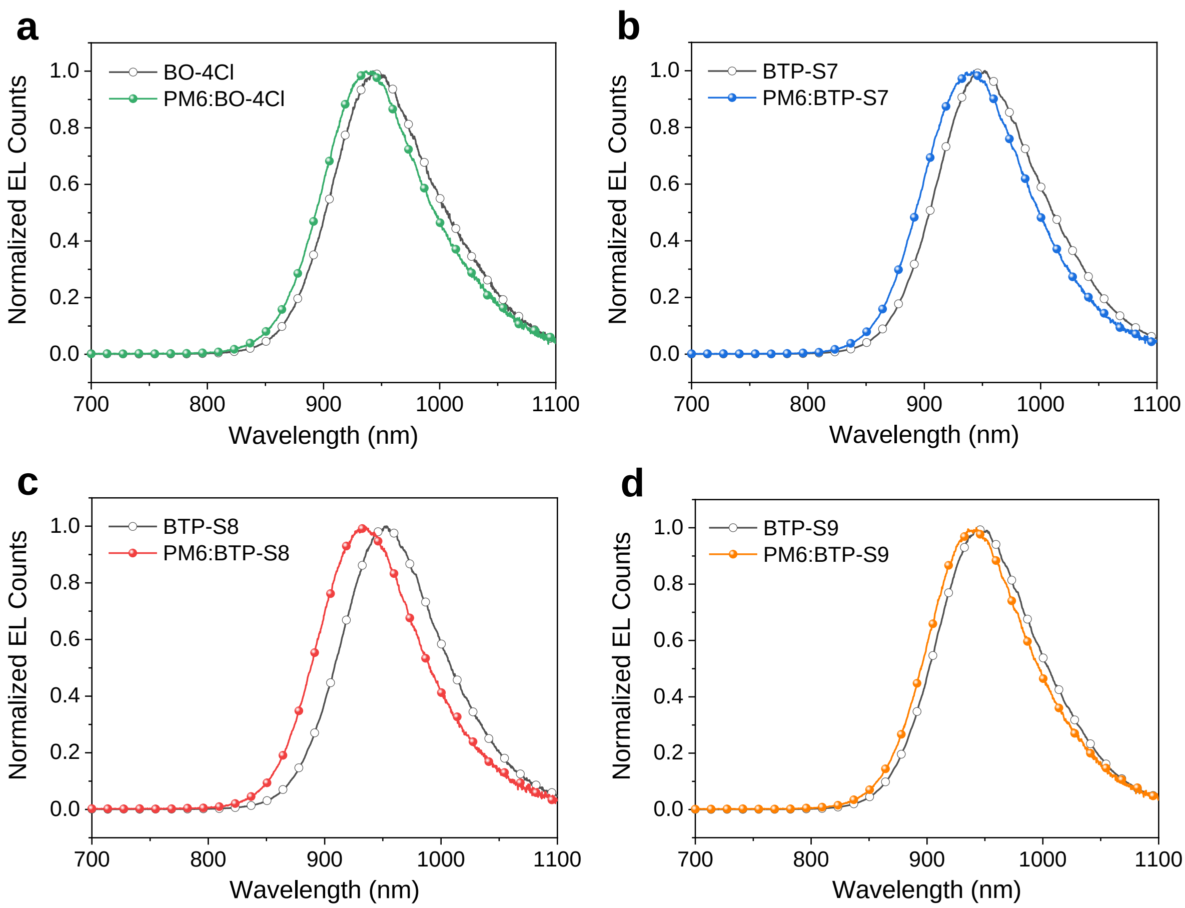

Supplementary Fig. 13 | a Normalized EL spectra of BO-4Cl and PM6:BO-4Cl films. **b** Normalized EL spectra of BTP-S7 and PM6:BTP-S7 films. **c** Normalized EL spectra of BTP-S8 and PM6:BTP-S8 films. **d** Normalized EL spectra of BTP-S9 and PM6:BTP-S9 films.

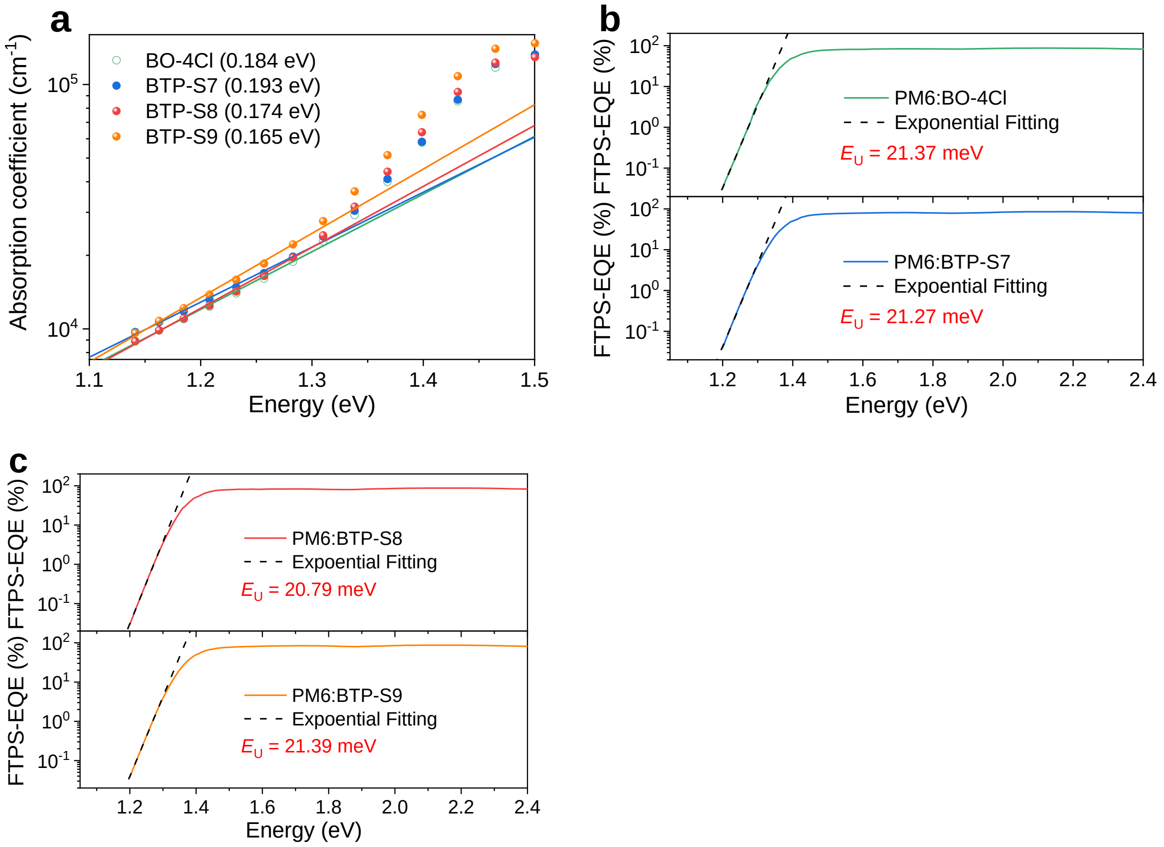

Supplementary Fig. 14 | a The calculation of Urbach energy (E_U) for BO-4Cl, BTP-S7, BTP-S8 and BTP-S9 neat films. **b** The calculation of Urbach energy (E_U) for PM6:BO-4Cl and PM6:BTP-S7-based OPVs. **c** The calculation of Urbach energy (E_U) for PM6:BTP-S8 and PM6:BTP-S9-based OPVs.

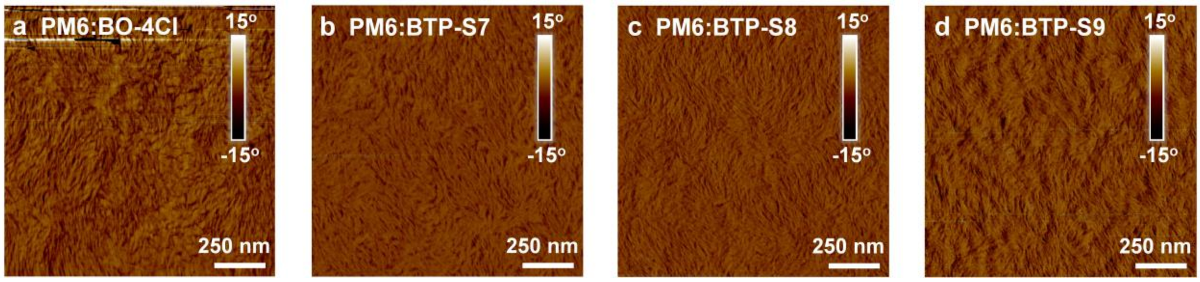

Supplementary Fig. 15 | AFM phase images for **a** PM6:BO4Cl, **b** PM6:BTP-S7, **c** PM6:BTP-S8 and **d** PM6:BTP-S9 blends.

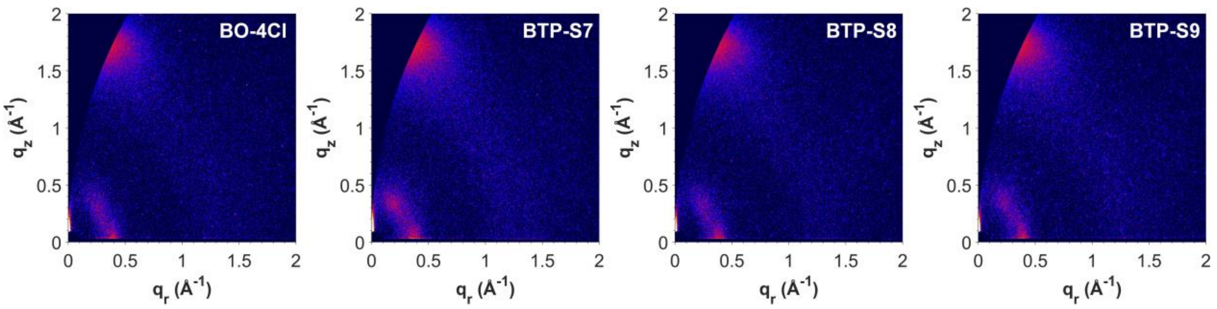

Supplementary Fig. 16 | 2D GIWAXS images for BO-4Cl, BTP-S7, BTP-S8 and BTP-S9 neat films.

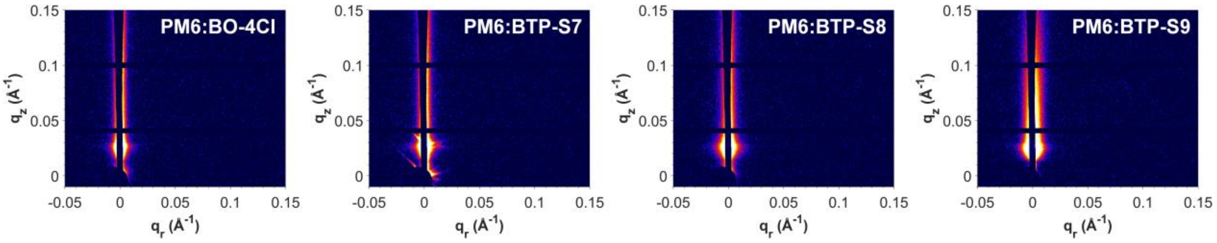

Supplementary Fig. 17 | 2D GISAXS images for PM6:BO-4Cl, PM6:BTP-S7, PM6:BTP-S8 and PM6:BTP-S9 blends.

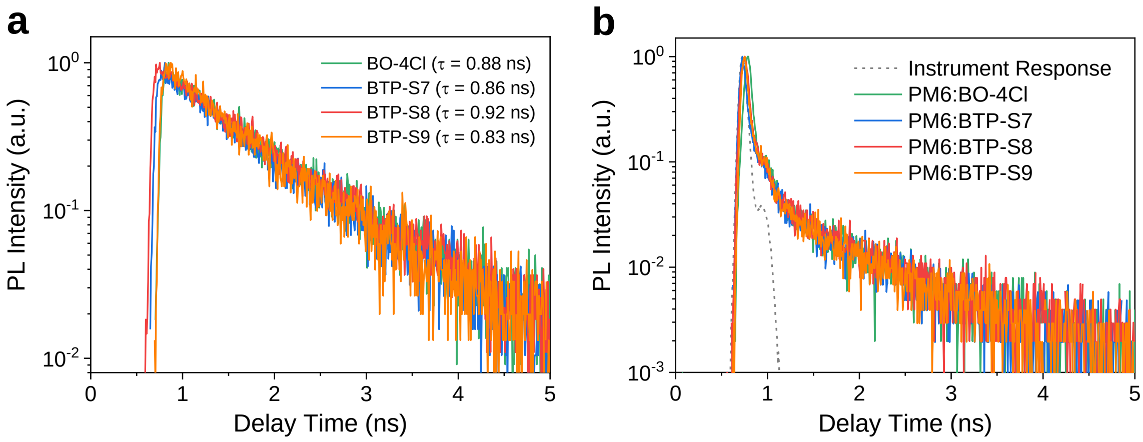

Supplementary Fig. 18 | a TRPL curves for BO-4Cl, BTP-S7, BTP-S8 and BTP-S9 neat films. **b** TRPL curves for PM6:BO-4Cl, PM6:BTP-S7, PM6:BTP-S8 and PM6:BTP-S9 blend films.

Supplementary Note 1

According to the formula of $L_D = \sqrt{D\tau}$, wherein L_D is the exciton diffusion length, D is the exciton diffusion constant (for efficient non-fullerene acceptors, e.g. Y6, it can be in the order of magnitude of 10^{-2} cm^2 s^{-1}, *J. Am. Chem. Soc.* 2019, *141*, 6922-6929), and τ is the exciton lifetime, the long exciton lifetime of 0.83 ns for BTP-S9 can allow an exciton diffusion length larger than 29 nm, leading to a tolerant pure acceptor domain size of 58 nm. So, the 53 nm pure acceptor domain size in PM6:BTP-S9 blend is still in the reasonable range for maintaining efficient hole transfer. So it is for BO-4Cl, BTP-S7 and BTP-S8 with even longer exciton lifetimes. Similar results were also observed in other reported works that a long carrier diffusion length of 41 nm was detected in PM6:Y6-based OPVs with an active layer thickness of 100 nm (*Adv. Energy Mater.* 2021, 2100804).

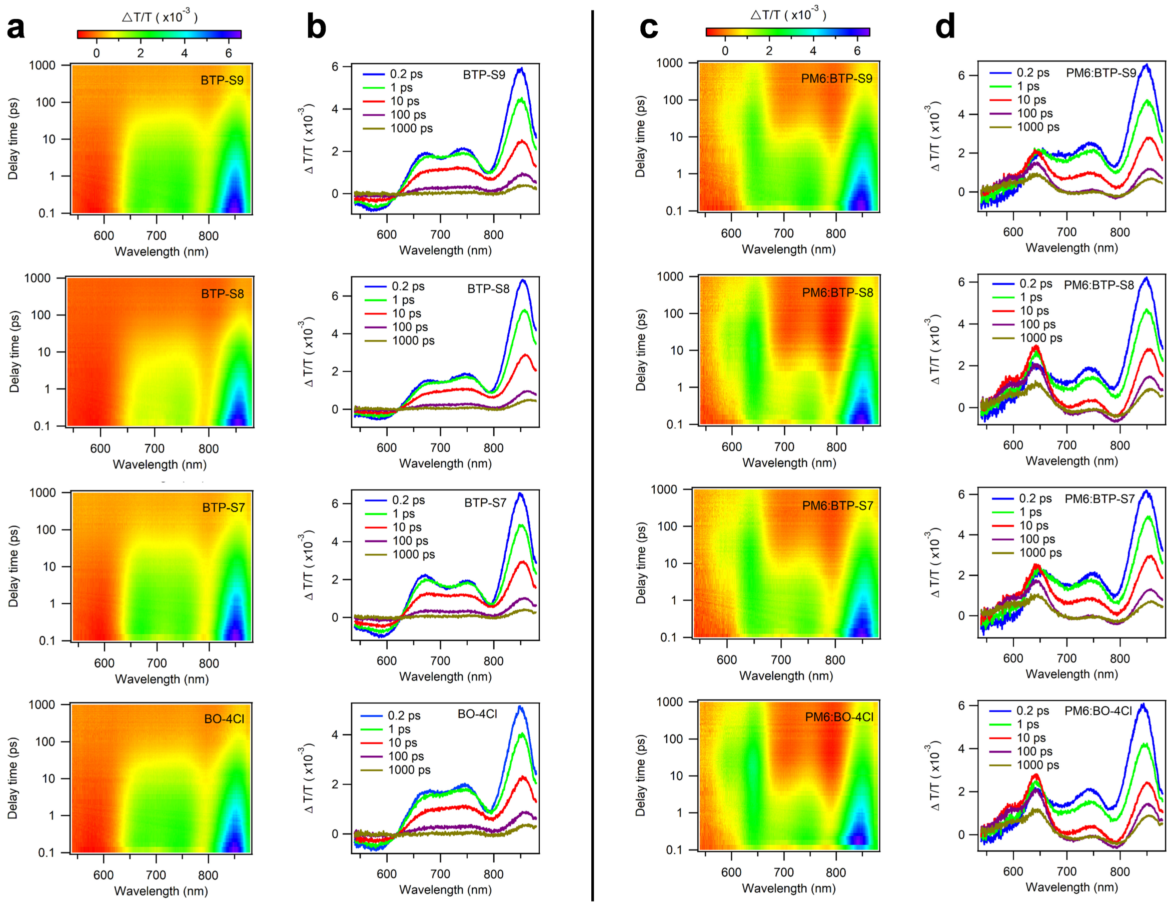

Supplementary Fig. 19 | a Color plot of TA spectra of BO-4Cl, BTP-S7, BTP-S8 and BTP-S9 neat films under 750 nm excitation. **b** Representative TA spectra at indicated delay times for pure acceptors. **c** Color plot of TA spectra of PM6:BO-4Cl, PM6:BTP-S7, PM6:BTP-S8 and PM6:BTP-S9 blend films under 750 nm excitation. **d** Representative TA spectra at indicated delay times for blend films.

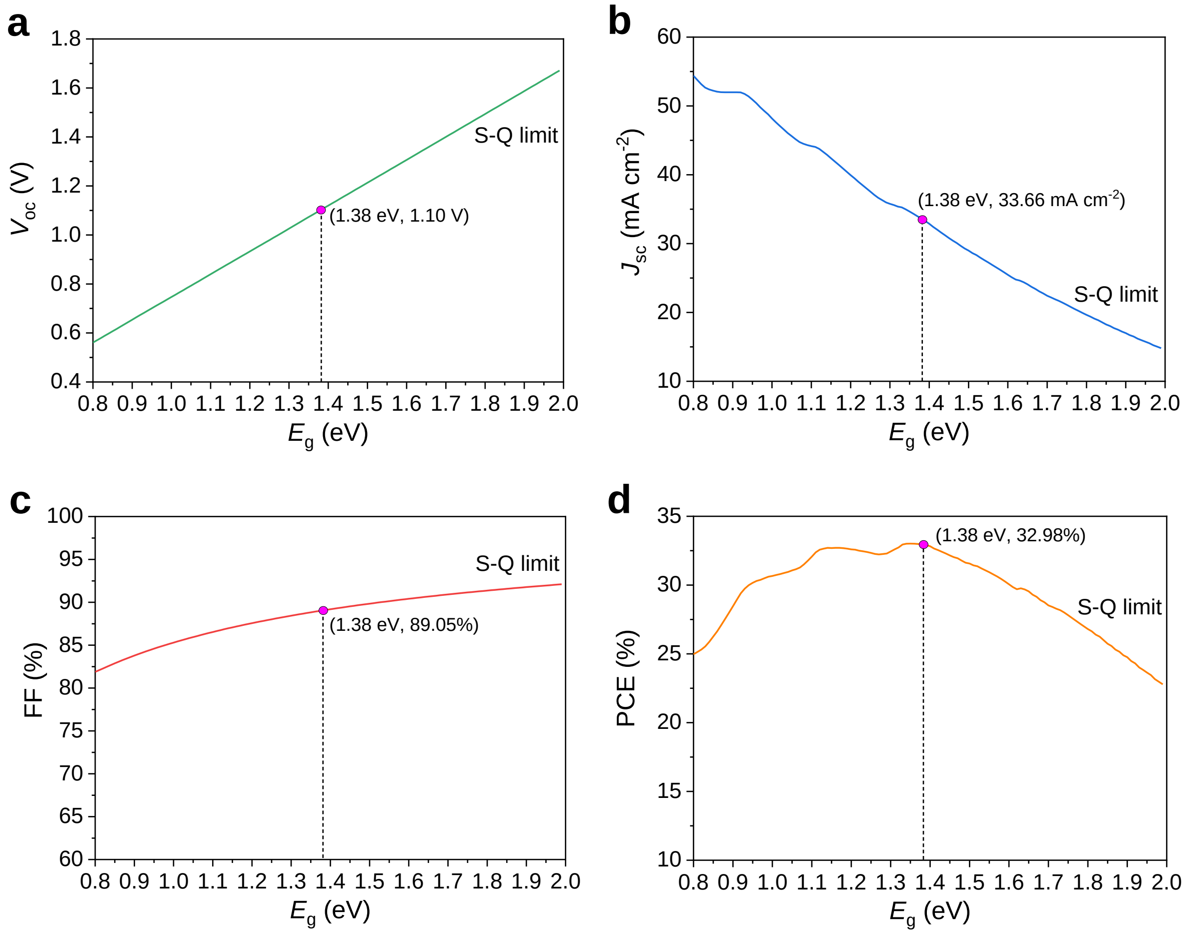

Supplementary Fig. 20 | a V_{oc} of S-Q limit at various bandgaps for solar cells under AM 1.5 G illumination. **b** J_{sc} of S-Q limit at various bandgaps for solar cells under AM 1.5 G illumination. **c** FF of S-Q limit at various bandgaps for solar cells under AM 1.5 G illumination. **d** PCE of S-Q limit at various bandgaps for solar cells under AM 1.5 G illumination. Raw data are summarized in **Supplementary Table 5**.

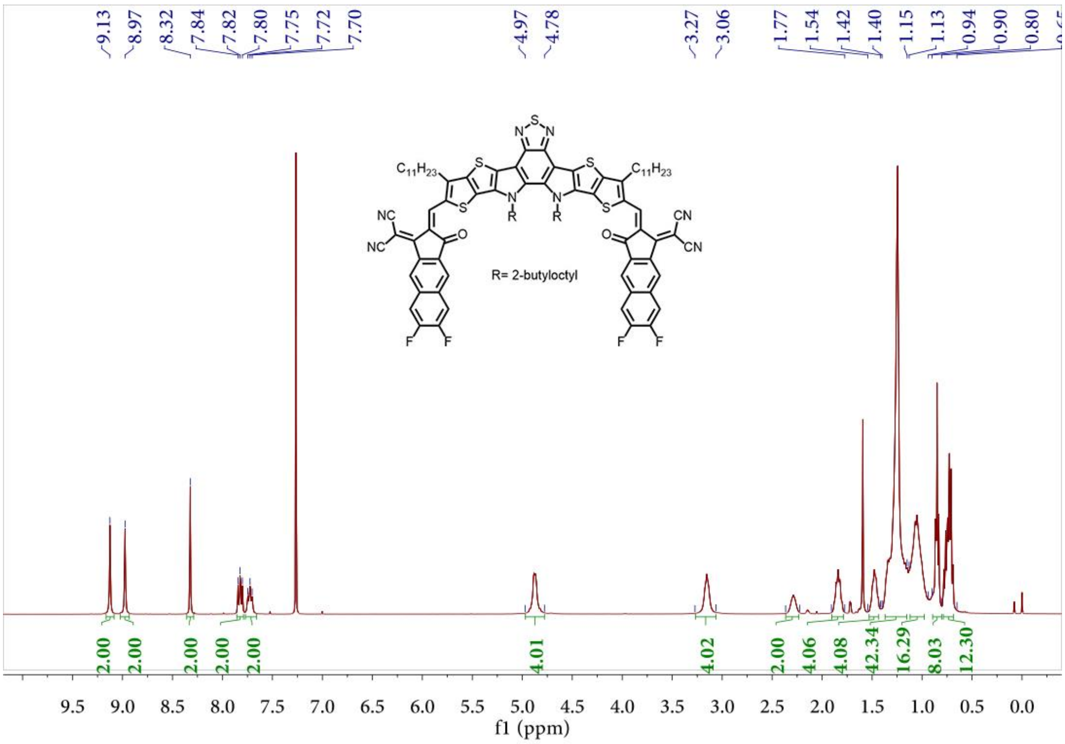

Supplementary Fig. 21 | ^{1}H NMR spectrum of **BTP-S7**.

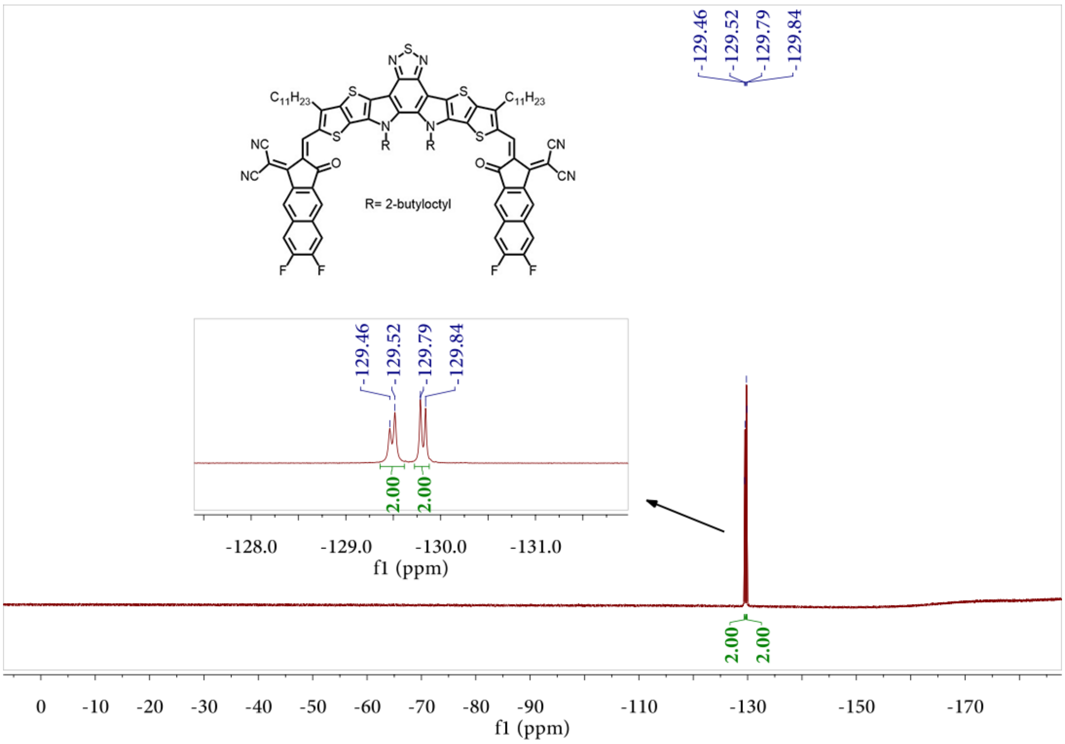

Supplementary Fig. 22 | ^{19}F NMR spectrum of **BTP-S7**.

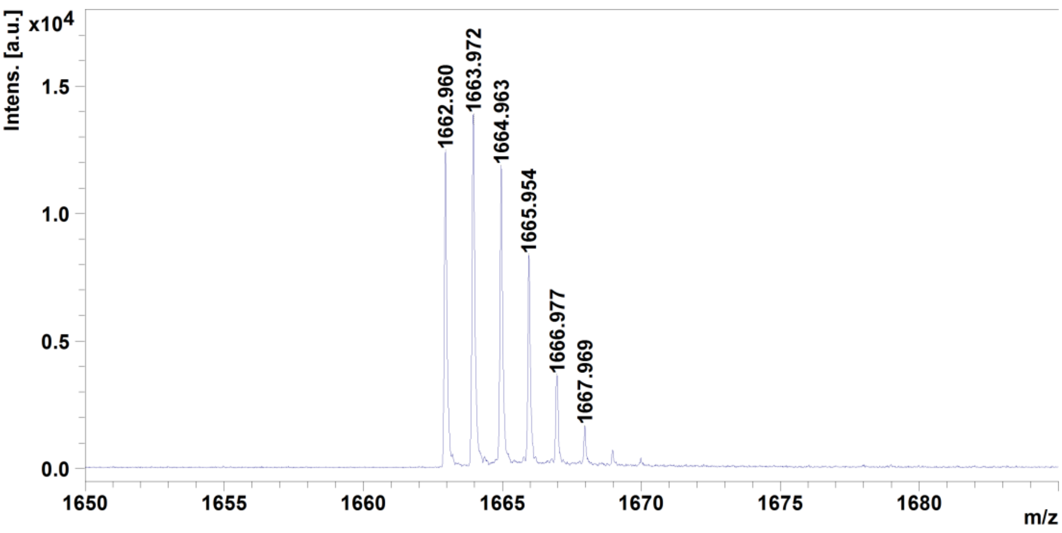

Supplementary Fig. 23 | MALDI-TOF mass spectrum of **BTP-S7**.

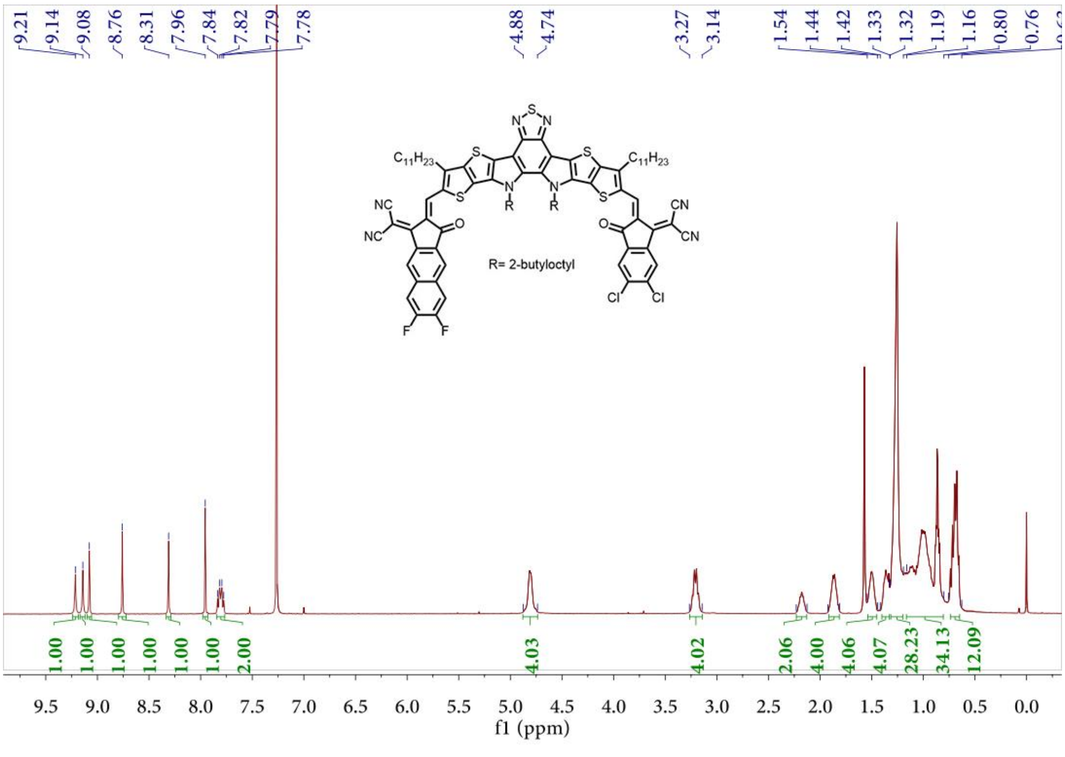

Supplementary Fig. 24 | ^{1}H NMR spectrum of **BTP-S8**.

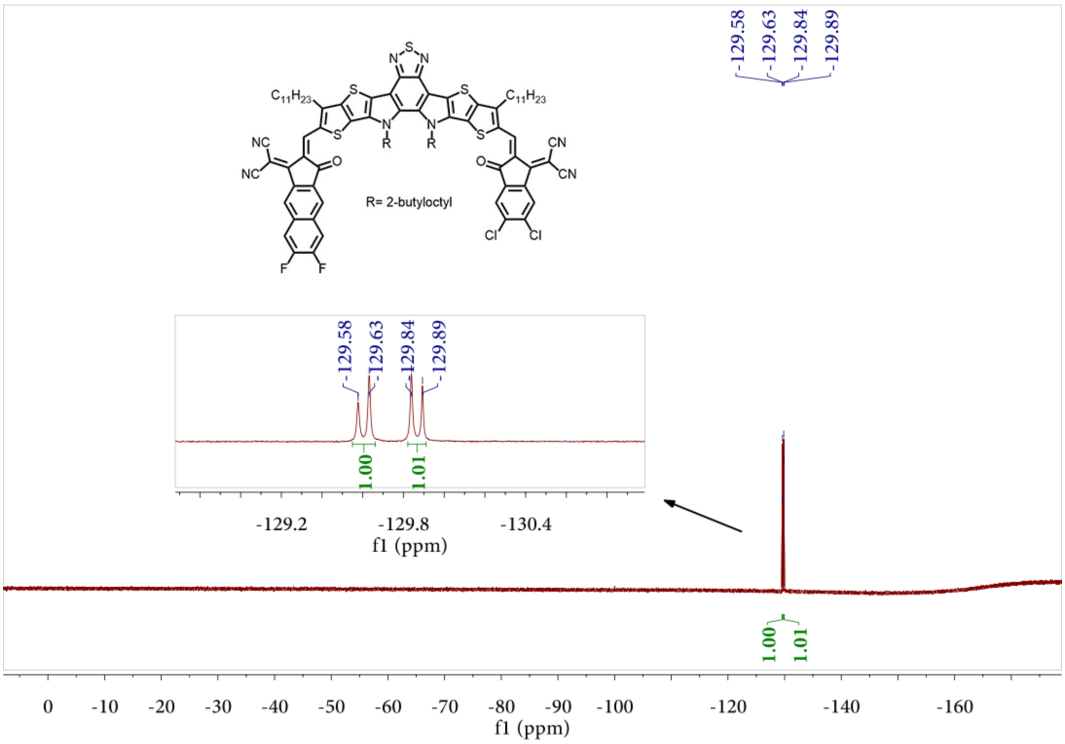

Supplementary Fig. 25 | ^{19}F NMR spectrum of **BTP-S8**.

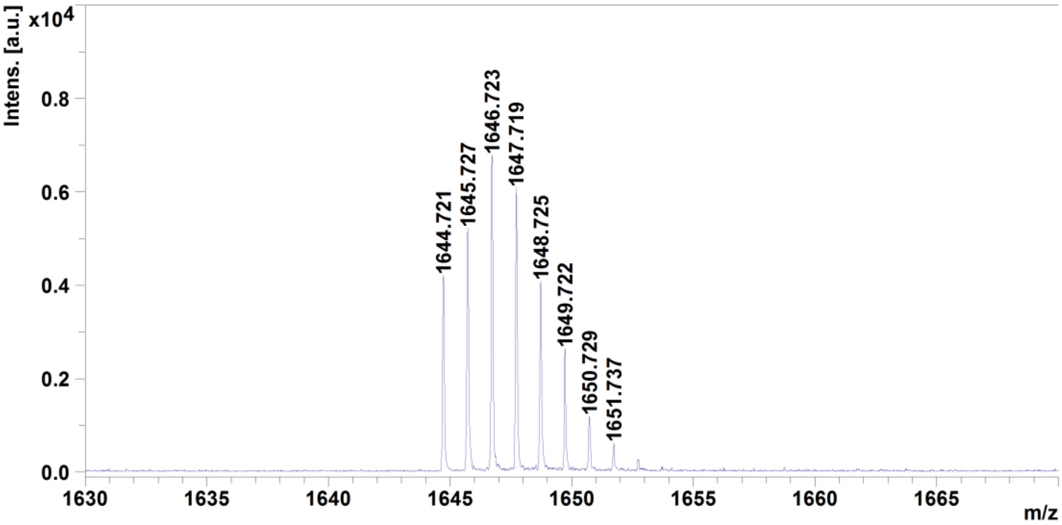

Supplementary Fig. 26 | MALDI-TOF mass spectrum of **BTP-S8**.

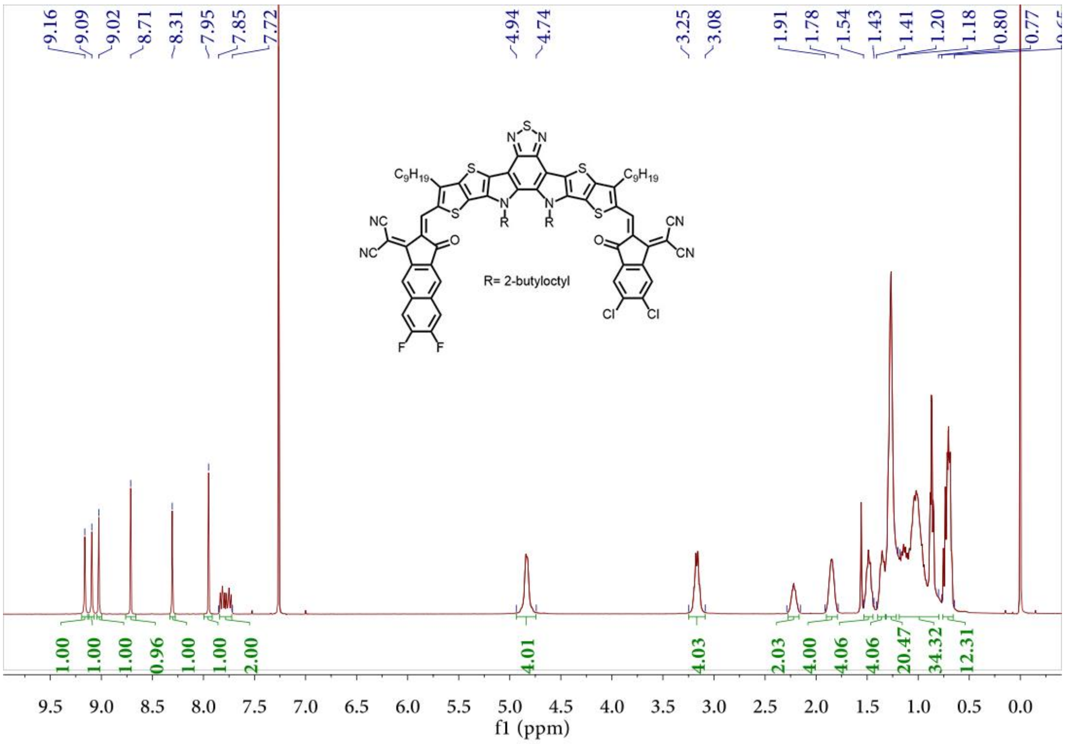

Supplementary Fig. 27 | ^{1}H NMR spectrum of **BTP-S9**.

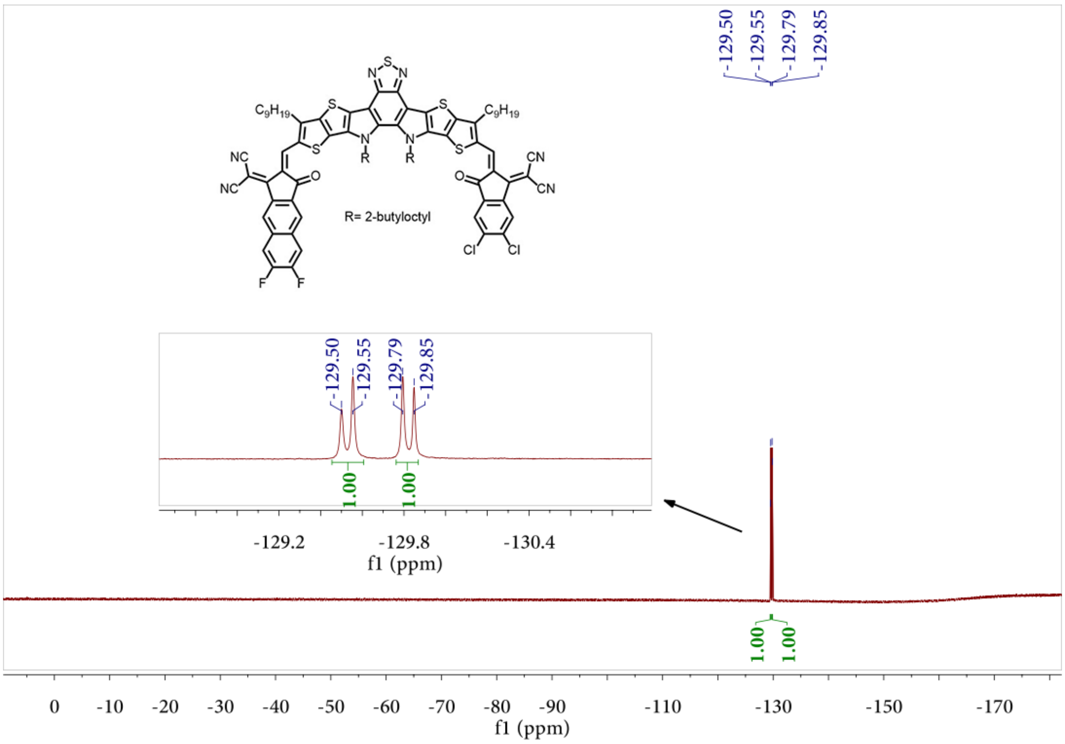

Supplementary Fig. 28 | ^{19}F NMR spectrum of **BTP-S9**.

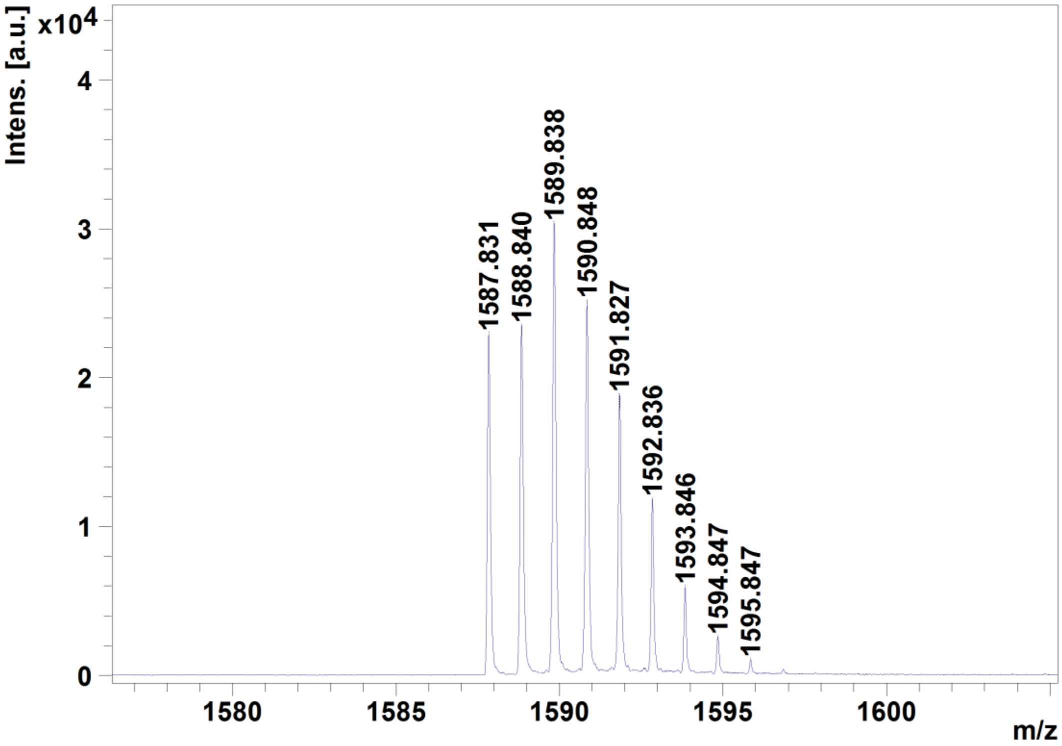

Supplementary Fig. 29 | MALDI-TOF mass spectrum of **BTP-S9**.

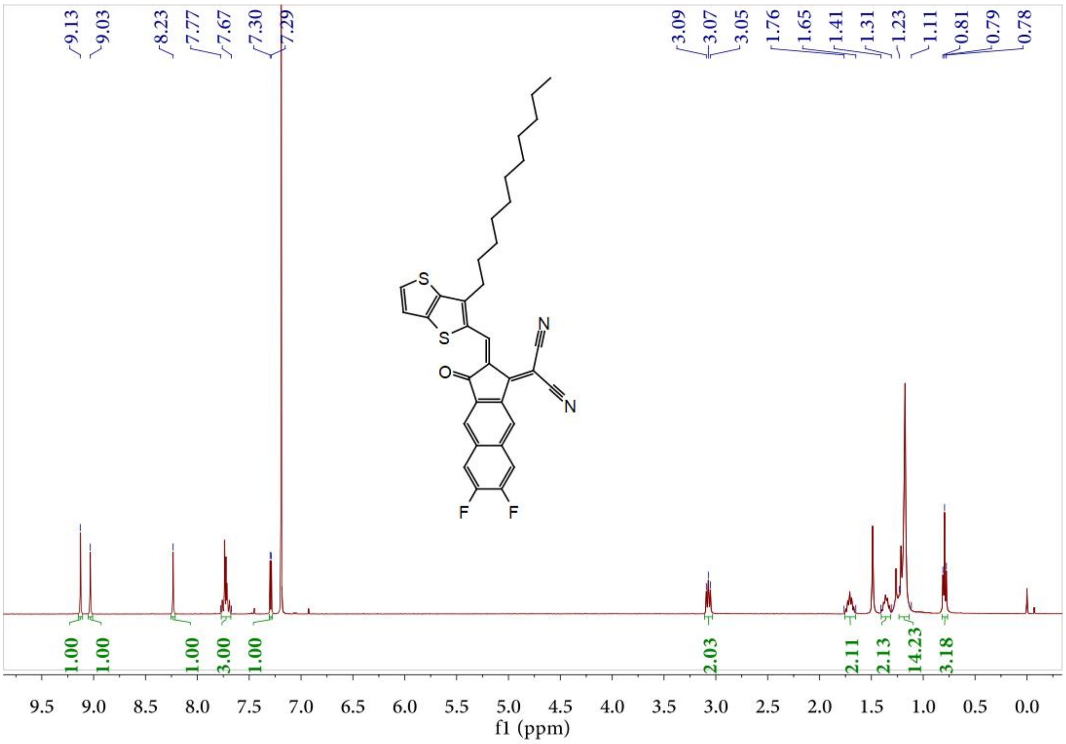

Supplementary Fig. 30 | ^{1}H NMR spectrum of **TNC**.

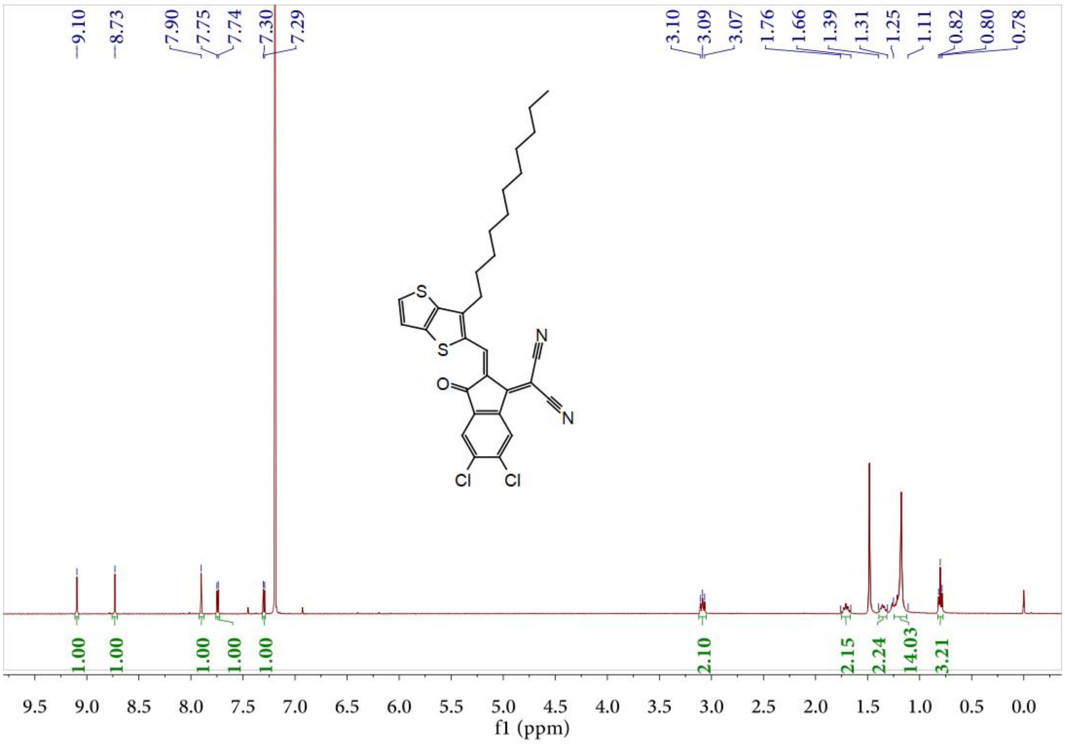

Supplementary Fig. 31 | ^{1}H NMR spectrum of **TIC**.

Supplementary Tables

Supplementary Table 1 Photovoltaic parameters of OPVs based on PM6:BTP-S7 blend under various optimization conditions.

PM6:BTP-S7	Additive	Annealing (°C)	V_{oc} (V)	J_{sc} (mA cm^{-2})	FF (%)	PCE (%)
1.2:1	w/o	As-cast	0.872	24.56	71.94	15.41
1:1	w/o	As-cast	0.866	24.57	72.89	15.51
1:1.2	w/o	As-cast	0.864	24.31	72.56	15.24
1:1	0.5% CN	As-cast	0.867	24.24	76.78	16.13
1:1	0.5% DIO	As-cast	0.865	24.73	74.34	15.90
1:1	0.5% CN	80	0.861	25.92	75.09	16.76
1:1	0.5% CN	100	0.855	24.99	76.53	16.39

Supplementary Table 2 Photovoltaic parameters of OPVs based on PM6:BTP-S8 blend under various optimization conditions.

PM6:BTP-S8	Additive	Annealing (°C)	V_{oc} (V)	J_{sc} (mA cm^{-2})	FF (%)	PCE (%)
1.2:1	w/o	As-cast	0.878	24.31	70.44	14.98
1:1	w/o	As-cast	0.871	25.89	70.83	15.93
1:1.2	w/o	As-cast	0.866	25.43	67.81	14.88
1:1	0.5% CN	As-cast	0.864	25.93	73.75	16.47
1:1	0.5% DIO	As-cast	0.853	20.69	74.25	13.06
1:1	0.3% CN	100	0.854	27.40	72.36	17.01
1:1	0.5% CN	100	0.852	26.96	75.45	17.33
1:1	0.8% CN	100	0.850	26.66	72.16	16.41

Supplementary Table 3 Photovoltaic parameters of OPVs based on PM6:BTP-S9 blend under various optimization conditions.

PM6:BTP-S9	Additive	Annealing (°C)	V_{oc} (V)	J_{sc} (mA cm^{-2})	FF (%)	PCE (%)
1.2:1	w/o	As-cast	0.873	24.60	74.17	15.97
1:1	w/o	As-cast	0.875	25.20	73.57	16.17
1:1.2	w/o	As-cast	0.873	26.02	73.12	16.56
1:1.5	w/o	As-cast	0.863	25.06	69.95	15.08
1:1.2	0.5% CN	100	0.847	25.68	72.21	15.75
1:1.2	0.25% DIO	100	0.846	26.06	78.07	17.20
1:1.2	0.5% DIO	100	0.841	25.82	76.97	16.79
1:1.2	0.25% DIO	70	0.855	26.36	77.03	17.31
1:1.2	0.25% DIO	80	0.846	26.47	78.44	17.56
1:1.2	0.25% DIO	90	0.852	26.16	77.51	17.23

Supplementary Table 4 Summary of photovoltaic parameters of binary OPVs based on asymmetric electron acceptors for this work and reported references.

Blend	V_{oc} (V)	J_{sc} (mA cm^{-2})	FF (%)	PCE (%)	Ref.
PM6:BTP-S8	0.852	26.96	75.45	17.33	This Work
PM6:BTP-S9	0.846	26.47	78.44	17.56	This Work
PM6:IDT6CN	0.830	15.14	73.77	9.27	1
PM6:IDT6CN-Th	0.810	16.75	76.72	10.41	1
PM6:IDT6CN-M	0.910	16.02	76.83	11.20	1
PBT1-C:TPTTIC	0.960	15.60	70.00	10.50	2
PBT1-C:TPTT-2F	0.881	15.82	73.00	10.17	3
PBT1-C:TPTTT-2F	0.916	17.63	74.50	12.03	3
PBDB-T:MeIC1	0.927	18.32	74.10	12.58	4
PBDB-T:IDT8CN-M	0.920	17.11	78.90	12.43	5
PBT1-C:TTPTTT-IC	0.996	12.47	63.70	7.91	6
PBT1-C:TTPTTT-2F	0.920	16.78	74.60	11.52	6
PBT1-C:TTPTTT-4F	0.863	19.36	72.10	12.05	6
PBT1-C:SePTT-2F	0.830	17.51	75.00	10.90	7
PBT1-C:SePTTT-2F	0.895	18.02	75.90	12.24	7

PBT1-C:TTPT-T-2F	0.915	18.50	75.10	12.71	8
PM6:a-IT	0.907	16.60	76.20	11.46	9
PM6:N7IT	0.932	21.04	70.50	13.82	9
PM6:N8IT	0.943	18.53	68.20	11.92	9
PBDB-T:a-BTTIC	0.904	20.31	74.00	13.60	10
PBDB-T:IPT-2F	0.860	22.40	72.40	14.00	11
PBDB-T:IPTT-2F	0.874	19.70	66.20	11.40	11
PBDB-T:IPTTT-2F	0.894	20.00	69.30	12.30	11
PBDB-T:IDT-OB	0.880	16.18	71.10	10.12	12
PBDB-T:IDTT-OB	0.910	16.58	74.00	11.19	13
PBT1-C-2Cl:IDTT-2F-Th	0.912	17.82	73.90	12.01	14
PBDB-TF:ITIC-2F	0.910	16.70	65.60	10.03	15
PBDB-TF:ITIC-3F	0.900	17.80	66.00	10.55	15
PBDB-T:*a*-IT-2OM	0.930	18.11	71.52	12.07	16
PBDB-T:*a*-IT-2F	0.780	19.06	68.84	10.28	16
PBDB-TF:IT-3F	0.900	20.35	75.50	13.83	17
J71:ZITI-3F	0.900	20.67	71.53	13.15	18
PM6:BDTP-4F	0.895	22.54	75.50	15.24	19
PM6:BTDTP-4F	0.866	21.25	71.30	13.12	19
PM6:BTN-4F	0.816	25.05	77.30	15.82	20
PM6:BTSe-4F	0.811	22.52	75.40	13.79	20
PM6:BTP-S1	0.934	22.39	72.69	15.21	21
PM6:BTP-S2	0.945	24.07	72.02	16.37	21
PM6:SY1	0.871	25.41	76.00	16.83	22
PM6:SY2	0.852	25.29	74.30	16.01	22
PM6:SY3	0.858	25.54	74.10	16.23	22
PM6:BP5T-4F	0.888	24.60	76.30	16.70	23
PM6:ABP4T-4F	0.922	22.00	75.10	15.20	23
PM6:BTIC-2Cl-gCF$_3$	0.840	25.09	76.99	16.31	24
PM6:BTP-2F-ThCl	0.869	25.38	77.40	17.06	25

Supplementary Table 5 Summary of E_g, V_{oc}, J_{sc}, FF and PCE parameters of S-Q limit for solar cells under AM 1.5G illumination.

E_g (eV)	V_{oc} (V)	J_{sc} (mA cm^{-2})	FF (%)	PCE (%)
0.8	0.5611	54.4006	81.8765	24.9741
0.81	0.5702	53.7593	82.0847	25.1425
0.82	0.5793	53.1511	82.2882	25.3170
0.83	0.5884	52.6660	82.4881	25.5443
0.84	0.5977	52.3919	82.6853	25.8731
0.85	0.6070	52.2260	82.8789	26.2549
0.86	0.6163	52.0709	83.0680	26.6402
0.87	0.6257	52.0071	83.2535	27.0731
0.88	0.6351	51.9833	83.4352	27.5278
0.89	0.6446	51.9820	83.6128	27.9954
0.9	0.6540	51.9818	83.7865	28.4642
0.91	0.6635	51.9815	83.9562	28.9336
0.92	0.6729	51.9773	84.1221	29.4012
0.93	0.6822	51.7556	84.2826	29.7391
0.94	0.6915	51.3970	84.4384	29.9901
0.95	0.7008	50.9197	84.5900	30.1620
0.96	0.7100	50.4049	84.7381	30.3025
0.97	0.7191	49.8053	84.8824	30.3804
0.98	0.7283	49.2843	85.0245	30.4986
0.99	0.7376	48.7723	85.1637	30.6138
1	0.7467	48.1760	85.2994	30.6644
1.01	0.7559	47.6213	85.4328	30.7327
1.02	0.7651	47.0838	85.5637	30.8032
1.03	0.7744	46.5677	85.6923	30.8790
1.04	0.7836	46.0661	85.8184	30.9561
1.05	0.7929	45.6209	85.9426	31.0644
1.06	0.8021	45.1627	86.0643	31.1556
1.07	0.8114	44.7564	86.1842	31.2764
1.08	0.8208	44.5025	86.3029	31.5020
1.09	0.8302	44.3155	86.4200	31.7729
1.1	0.8397	44.1662	86.5351	32.0690
1.11	0.8492	44.0460	86.6484	32.3848
1.12	0.8585	43.7638	86.7586	32.5741
1.13	0.8678	43.3390	86.8658	32.6476
1.14	0.8771	42.9026	86.9711	32.7046
1.15	0.8864	42.3916	87.0740	32.6943
1.16	0.8956	41.9178	87.1755	32.7052
1.17	0.9049	41.4391	87.2751	32.7036
1.18	0.9142	40.9476	87.3730	32.6830
1.19	0.9234	40.4472	87.4692	32.6463

1.2	0.9327	39.9386	87.5637	32.5939
1.21	0.9420	39.4816	87.6571	32.5763
1.22	0.9512	38.9651	87.7484	32.4995
1.23	0.9605	38.4972	87.8387	32.4555
1.24	0.9697	38.0296	87.9275	32.4036
1.25	0.9790	37.5525	88.0148	32.3349
1.26	0.9883	37.0828	88.1007	32.2643
1.27	0.9976	36.6636	88.1857	32.2309
1.28	1.0069	36.3204	88.2697	32.2595
1.29	1.0163	35.9896	88.3526	32.2934
1.3	1.0258	35.7845	88.4350	32.4385
1.31	1.0353	35.5979	88.5163	32.5974
1.32	1.0447	35.3892	88.5962	32.7320
1.33	1.0542	35.2710	88.6754	32.9497
1.34	1.0637	34.9888	88.7525	33.0065
1.35	1.0730	34.6617	88.8282	33.0144
1.36	1.0824	34.3244	88.9027	33.0064
1.37	1.0918	33.9764	88.9761	32.9817
1.38	1.1012	33.6594	89.0486	32.9818
1.39	1.1105	33.2819	89.1197	32.9147
1.4	1.1198	32.8860	89.1897	32.8221
1.41	1.1291	32.4241	89.2584	32.6541
1.42	1.1384	32.0241	89.3264	32.5423
1.43	1.1477	31.6206	89.3934	32.4196
1.44	1.1571	31.2236	89.4596	32.2964
1.45	1.1664	30.8140	89.5248	32.1525
1.46	1.1757	30.4318	89.5892	32.0306
1.47	1.1851	30.0904	89.6530	31.9461
1.48	1.1944	29.6826	89.7156	31.7828
1.49	1.2037	29.2873	89.7774	31.6257
1.5	1.2131	28.9855	89.8389	31.5657
1.51	1.2224	28.6238	89.8993	31.4328
1.52	1.2318	28.3309	89.9592	31.3713
1.53	1.2411	27.9562	90.0179	31.2110
1.54	1.2505	27.6063	90.0760	31.0724
1.55	1.2598	27.2591	90.1334	30.9305
1.56	1.2691	26.9094	90.1900	30.7794
1.57	1.2785	26.5595	90.2459	30.6216
1.58	1.2878	26.2008	90.3011	30.4469
1.59	1.2971	25.8350	90.3555	30.2572
1.6	1.3064	25.4675	90.4092	30.0588
1.61	1.3157	25.1015	90.4622	29.8555
1.62	1.3251	24.7803	90.5149	29.7002
1.63	1.3346	24.6394	90.5679	29.7613

1.64	1.3441	24.3911	90.6197	29.6864
1.65	1.3534	24.0915	90.6706	29.5427
1.66	1.3627	23.7186	90.7205	29.3013
1.67	1.3721	23.4187	90.7701	29.1455
1.68	1.3814	23.0558	90.8188	28.9037
1.69	1.3907	22.7678	90.8674	28.7517
1.7	1.4000	22.4268	90.9151	28.5255
1.71	1.4095	22.1776	90.9628	28.4131
1.72	1.4189	21.9166	91.0098	28.2805
1.73	1.4283	21.6834	91.0565	28.1799
1.74	1.4376	21.3987	91.1023	28.0064
1.75	1.4470	21.0988	91.1475	27.8072
1.76	1.4563	20.8004	91.1922	27.6043
1.77	1.4657	20.5073	91.2364	27.4033
1.78	1.4750	20.2167	91.2802	27.2002
1.79	1.4844	19.9295	91.3235	26.9964
1.8	1.4937	19.6540	91.3663	26.8037
1.81	1.5031	19.4015	91.4089	26.6379
1.82	1.5124	19.0971	91.4506	26.3944
1.83	1.5218	18.8680	91.4923	26.2519
1.84	1.5311	18.5621	91.5332	25.9957
1.85	1.5404	18.2571	91.5735	25.7351
1.86	1.5498	18.0268	91.6139	25.5768
1.87	1.5591	17.7240	91.6534	25.3088
1.88	1.5685	17.5022	91.6930	25.1537
1.89	1.5778	17.2150	91.7318	24.8981
1.9	1.5872	17.0066	91.7706	24.7540
1.91	1.5965	16.7116	91.8085	24.4769
1.92	1.6059	16.4947	91.8465	24.3114
1.93	1.6152	16.1951	91.8835	24.0172
1.94	1.6245	15.9728	91.9207	23.8347
1.95	1.6339	15.7469	91.9574	23.6425
1.96	1.6433	15.5276	91.9939	23.4564
1.97	1.6525	15.2416	92.0295	23.1630
1.98	1.6619	15.0283	92.0652	22.9774
1.99	1.6713	14.8161	92.1005	22.7895

Supplementary Methods

Materials. Raw materials and solvents were purchased from commercial companies and used without further purifications. **PM6** and **BO-4Cl** were purchased from Solarmer Materials Inc. Non-fullerene acceptors of **BTP-S7**, **BTP-S8**, **BTP-S9** and molecular fragments of **TNC**, **TIC** were synthesized according to the procedures described below.

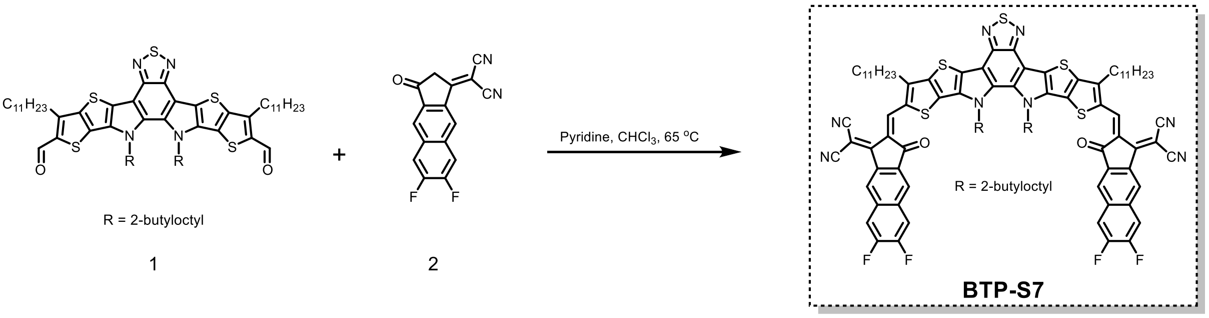

2,2'-((2Z,2'Z)-((12,13-bis(2-butyloctyl)-3,9-diundecyl-12,13-dihydro-[1,2,5]thiadiazolo[3,4-e]thieno[2",3":4',5']thieno[2',3':4,5]pyrrolo[3,2-g]thieno[2',3':4,5]thieno[3,2-b]indole-2,10-diyl)bis(methanylylidene))bis(6,7-difluoro-3-oxo-2,3-dihydro-1H-cyclopenta[b]naphthalene-2,1-diylidene))dimalononitrile (**BTP-S7**)

To a Schlenk tube were added **Compound 1** (0.42 g, 0.38 mmol), **Compound 2** (0.45 g, 1.6 mmol) and $CHCl_3$ (60 mL). The mixture was frozen with liquid nitrogen, followed by three times of successive vacuum and nitrogen fill cycles. Under the protection of N_2, 1 mL pyridine was injected. Then, the reactant was refluxed at 65 °C for 18 h. The crude product was purified using silica gel column chromatography with the mixture of petroleum ether and dichloromethane (1:2, v/v) as the eluent, yielding a black solid (0.54 g, 85%). ^{1}H NMR (400 MHz, $CDCl_3$): δ = 9.13 (s, 2H), 8.97 (s, 2H), 8.32 (s, 2H), 7.86-7.79 (m, 2H), 7.72 (t, *J* = 8.7 Hz, 2H), 4.97-4.78 (m, 4H), 3.27-3.06 (m, 4H), 2.37-2.22 (m, 2H), 1.91-1.77 (m, 4H), 1.54-1.42 (m, 4H), 1.40-1.15 (m, 42H), 1.13-0.94 (m, 16H), 0.85 (t, *J* = 6.7 Hz, 8H), 0.79-0.65 (m, 12H). ^{19}F NMR (400 MHz, $CDCl_3$): δ = -129.49 (d, *J* = 20.4 Hz, 2F), -129.81 (d, *J* = 20.5 Hz, 2F). MS (MALDI-TOF): Cald for $C_{98}H_{106}F_4N_8O_2S_5$ (M^+): 1664.27, Found: 1663.97.

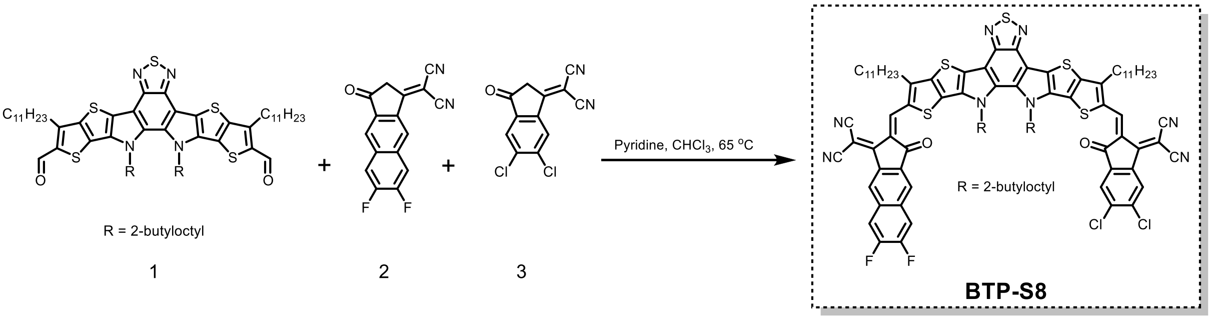

2-((Z)-2-((12,13-bis(2-butyloctyl)-10-(((Z)-5,6-dichloro-1-(dicyanomethylene)-3-oxo-1,3-dihydro-2H-inden-2-ylidene)methyl)-3,9-diundecyl-12,13-dihydro-[1,2,5]thiadiazolo[3,4-e]thieno[2",3":4',5']thieno[2',3':4,5]pyrrolo[3,2-g]thieno[2',3':4,5]thieno[3,2-b]indol-2-

yl)methylene)-6,7-difluoro-3-oxo-2,3-dihydro-1H-cyclopenta[b]naphthalen-1-ylidene)malononitrile (**BTP-S8**)

To a Schlenk tube were added **Compound 1** (0.56 g, 0.5 mmol), **Compound 2** (0.28 g, 1 mmol), **Compound 3** (0.26 g, 1 mmol) and $CHCl_3$ (50 mL). The mixture was frozen with liquid nitrogen, followed by three times of successive vacuum and nitrogen fill cycles. Under the protection of N_2, 1 mL pyridine was injected. Then, the reactant was refluxed at 65 °C for 16 h. The crude product was purified using silica gel column chromatography with the mixture of petroleum ether and dichloromethane (1:1, v/v) as the eluent, yielding a black solid (0.3 g, 36%). ^{1}H NMR (400 MHz, $CDCl_3$): δ = 9.21 (s, 1H), 9.14 (s, 1H), 9.08 (s, 1H), 8.76 (s, 1H), 8.31 (s, 1H), 7.96 (s, 1H), 7.81 (dd, *J* = 16.4, 7.3 Hz, 2H), 4.88-4.74 (m, 4H), 3.27-3.14 (m, 4H), 2.23-2.13 (m, 2H), 1.93-1.81 (m, 4H), 1.54-1.44 (m, 4H), 1.42-1.33 (m, 4H), 1.32-1.19 (m, 28H), 1.16-0.80 (m, 34H), 0.76-0.63 (m, 12H). ^{19}F NMR (400 MHz, $CDCl_3$): δ = -129.60 (d, *J* = 20.4 Hz, 1F), -129.87 (d, *J* = 20.5 Hz, 1F). MS (MALDI-TOF): Cald for $C_{94}H_{104}Cl_2F_2N_8O_2S_5$ (M^+): 1647.12, Found: 1646.72.

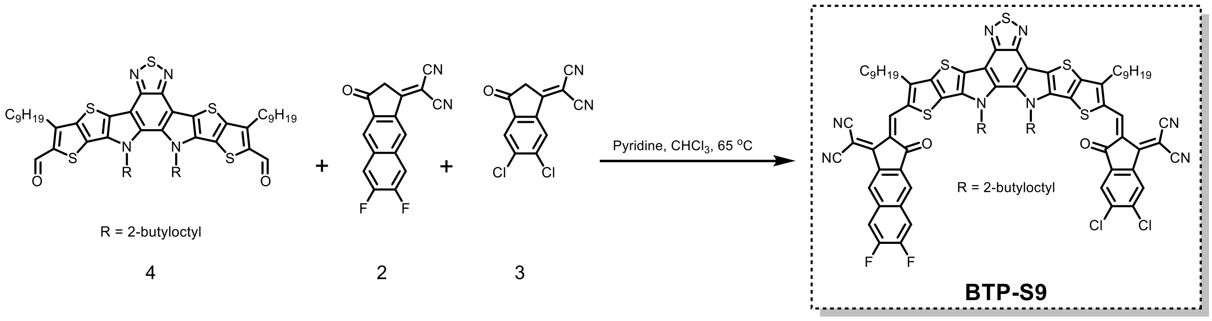

2-((Z)-2-((12,13-bis(2-butyloctyl)-10-(((Z)-5,6-dichloro-1-(dicyanomethylene)-3-oxo-1,3-dihydro-2H-inden-2-ylidene)methyl)-3,9-dinonyl-12,13-dihydro-[1,2,5]thiadiazolo[3,4-e]thieno[2'',3'':4',5']thieno[2',3':4,5]pyrrolo[3,2-g]thieno[2',3':4,5]thieno[3,2-b]indol-2-yl)methylene)-6,7-difluoro-3-oxo-2,3-dihydro-1H-cyclopenta[b]naphthalen-1-ylidene)malononitrile (**BTP-S9**)

To a Schlenk tube were added **Compound 4** (0.53 g, 0.49 mmol), **Compound 2** (0.28 g, 1 mmol), **Compound 3** (0.26 g, 1 mmol) and $CHCl_3$ (50 mL). The mixture was frozen with liquid nitrogen, followed by three times of successive vacuum and nitrogen fill cycles. Under the protection of N_2, 1 mL pyridine was injected. Then, the reactant was refluxed at 65 °C for 20 h. The crude product was purified using silica gel column chromatography with the mixture of petroleum ether and dichloromethane (1:1, v/v) as the eluent, yielding a black solid (0.28 g, 36%). ^{1}H NMR (400 MHz, $CDCl_3$): δ = 9.16 (s, 1H), 9.09 (s, 1H), 9.02 (s, 1H), 8.71 (s, 1H), 8.31 (s, 1H), 7.95 (s, 1H), 7.85-7.72 (m, 2H), 4.94-4.74 (m, 4H), 3.25-3.08 (m, 4H), 2.29-2.15 (m, 2H), 1.91-1.78 (m, 4H), 1.54-1.43 (m, 4H), 1.41-1.32 (m, 4H), 1.31-1.20 (m, 20H), 1.18-0.80 (m, 34H), 0.77-0.65 (m, 12H). ^{19}F NMR (400 MHz, $CDCl_3$): δ = -129.53 (d, *J* = 20.5 Hz, 1F), -129.82 (d, *J* = 20.5 Hz, 1F). MS (MALDI-TOF): Cald for $C_{90}H_{96}Cl_2F_2N_8O_2S_5$ (M^+): 1591.01, Found: 1590.85.

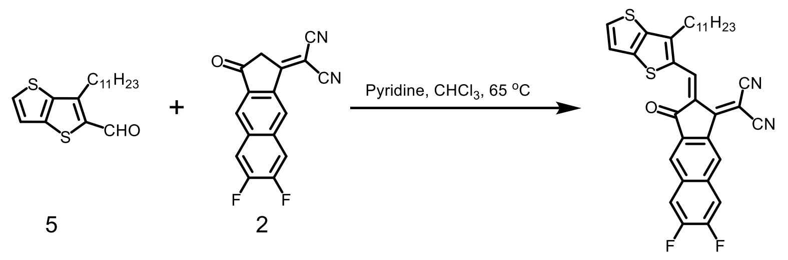

(Z)-2-(6,7-difluoro-3-oxo-2-((3-undecylthieno[3,2-b]thiophen-2-yl)methylene)-2,3-dihydro-1H-cyclopenta[b]naphthalen-1-ylidene)malononitrile (**TNC**)

To a Schlenk tube were added **Compound 5** (0.097 g, 0.3 mmol), **Compound 2** (0.14 g, 0.5 mmol) and $CHCl_3$ (30 mL). The mixture was frozen with liquid nitrogen, followed by three times of successive vacuum and nitrogen fill cycles. Under the protection of N_2, 0.6 mL pyridine was injected. Then, the reactant was refluxed at 65 °C for 4 h. The crude product was purified using silica gel column chromatography with the mixture of petroleum ether and dichloromethane (1:2, v/v) as the eluent, yielding a red solid (0.07 g, 40%). ^{1}H NMR (400 MHz, $CDCl_3$): δ = 9.13 (s, 1H), 9.03 (s, 1H), 8.23 (s, 1H), 7.77-7.67 (m, 3H), 7.29 (d, *J* = 5.2 Hz, 1H), 3.07 (t, *J* = 7.8 Hz, 2H), 1.76-1.65 (m, 2H), 1.41-1.31 (m, 2H), 1.23-1.11 (m, 14H), 0.79 (t, *J* = 6.8 Hz, 3H).

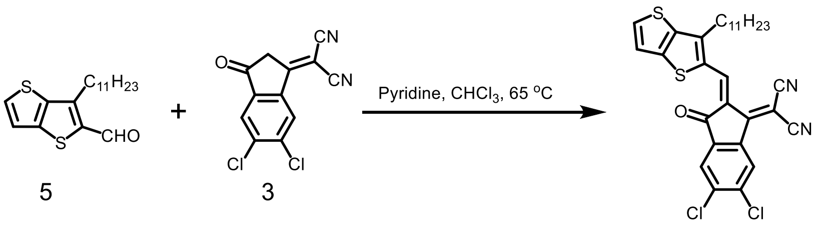

(Z)-2-(5,6-dichloro-3-oxo-2-((3-undecylthieno[3,2-b]thiophen-2-yl)methylene)-2,3-dihydro-1H-inden-1-ylidene)malononitrile (**TIC**)

To a Schlenk tube were added **Compound 5** (0.097 g, 0.3 mmol), **Compound 3** (0.13 g, 0.5 mmol) and $CHCl_3$ (30 mL). The mixture was frozen with liquid nitrogen, followed by three times of successive vacuum and nitrogen fill cycles. Under the protection of N_2, 0.6 mL pyridine was injected. Then, the reactant was refluxed at 65 °C for 4 h. The crude product was purified using silica gel column chromatography with the mixture of petroleum ether and dichloromethane (1:2, v/v) as the eluent, yielding a brown solid (0.08 g, 47%). ^{1}H NMR (400 MHz, $CDCl_3$): δ = 9.10 (s, 1H), 8.73 (s, 1H), 7.90 (s, 1H), 7.74 (d, *J* = 5.2 Hz, 1H), 7.30 (d, *J* = 5.2 Hz, 1H), 3.09 (t, J = 7.7 Hz, 2H), 1.76-1.66 (m, 2H), 1.39-1.31 (m, 2H), 1.25-1.11 (m, 14H), 0.80 (t, *J* = 6.8 Hz, 3H).

General Characterizations. ^{1}H NMR and ^{19}F NMR spectra were obtained on a Bruker Advance III 400 (400 MHz) nuclear magnetic resonance (NMR) spectroscope. MALDI-TOF

MS spectra were measured on a Walters Maldi Q-TOF Premier mass spectrometry. UV-vis-NIR absorption spectra were recorded on a Shimadzu UV-1800 spectrophotometer. Cyclic voltammetry (CV) was done on a CHI600A electrochemical workstation with Pt disk, Pt plate, and standard calomel electrode (SCE) as working electrode, counter electrode, and reference electrode, respectively, in a 0.1 mol/L tetrabutylammoniumhexafluorophosphate (Bu_4NPF_6) acetonitrile solution. The CV curves were recorded versus the potential of SCE, which was calibrated by the ferrocene-ferrocenium (Fc/Fc^+) redox couple (4.8 eV below the vacuum level). The equation of $E_{LUMO/HOMO}$ = -e($E_{red/ox}$+4.41) (eV) was used to calculate the LUMO and HOMO levels (the redox potential of Fc/Fc^+ is found to be 0.39 V). AFM images were obtained on a VeecoMultiMode atomic force microscopy in the tapping mode.

Mobility Measurement. The charge carrier mobilities of the blend films were measured using the space-charge-limited current (SCLC) method. Hole-only devices were fabricated in a structure of ITO/PEDOT:PSS/Active Layer/MoO_3/Ag, electron-only devices were fabricated in a structure of ITO/ZnO/Active Layer/PFN-Br/Ag. The device characteristics were extracted by modeling the dark current under forward bias using the SCLC expression described by the Mott-Gurney law:

$$J = \frac{9}{8}\varepsilon_r \varepsilon_0 \mu \frac{V^2}{L^3} \qquad (1)$$

Here, $\varepsilon_r \approx 3$ is the average dielectric constant of the blend film, ε_0 is the permittivity of the free space, μ is the carrier mobility, L is the thickness of the film, and V is the applied voltage.

Electroluminescence Measurement. An external current/voltage source was employed to provide an external electric field to the pristine and blended solar cells. The electroluminescence emissions were recorded with an Andor spectrometer.

FTPS-EQE Measurement. The FTPS-EQE was measured with a Vertex 70 from Bruker Optics, which was equipped with a quartz tungsten halogen lamp, quartz beam-splitter and external detector option. A low-noise current amplifier (SR570) was used to amplify the photocurrent produced under illumination of the solar cells, with light modulated by the Fourier transform infrared spectroscope (FTIR). The output voltage of the current amplifier was fed back into the external detector port of the FTIR to use the FTIR software to collect the photocurrent spectra.

EQE_{EL} Measurement. The EQE_{EL} was recorded with an in-house-built system comprising a Hamamatsu silicon photodiode 1010B, Keithley 2400 source meter (for supplying voltages and recording injected currents), and Keithley 485 picoammeter (for measuring the emitted light intensity).

GIWAXS/GISAXS. GIWAXS/GISAXS measurements with Kα X-ray of Cu source (8.05 keV, 1.54 Å) and a Pilatus3R 300 K detector were conducted at a Xeuss 2.0 SAXS/WAXS laboratory beamline. Samples were prepared by spin coating identical chloroform blend solutions as those used in OPVs on Si substrates. The grazing incident angle were 0.2°.

Time-Resolved Photoluminescence Spectroscopy (TRPL). The optically pumped lasing measurements were taken on a home-build far-field microfluorescence system (Olympus, IX73 inverted microscope). The crystal sample was immersed by diethylether and then dispersed onto a glass substrate. The excitation light (515 nm) was generated from the second harmonic of the fundamental output that was seeded by a mode-locked Ti:sapphire laser (Light Conversion Pharos, 1030 nm, < 300 fs, 1 MHz). The excitation light was filtered with a 515 nm band-pass filter and then diverged with a convex lens (f = 500 mm), finally focused down to a 140 μm diameter spot through an objective lens (Olympus MplanFLN, 20x, NA = 0.45). The emission light was collected by the same objective and focused into a spectrograph (Princeton Instruments, Acton SpectraPro, SP-2300i) with a 600 mm^{-1} grating and detected by a liquid-N_2-cooled CCD (PyLon 100B excelon). The instrument resolution (FWHM) was ~0.1 nm. All measurements were taken at room temperature with pulse picker = 1000. TRPL decay kinetics were collected using a TCSPC module (PicoHarp 300) and a SPAD detector (IDQ, id100) with an instrument response function ~100 ps. The two-photon pumped lasing performance was measured upon excitation at 1030 nm.

Transient Absorption Spectroscopy (TAS) Measurement. For femtosecond transient absorption spectroscopy, the fundamental output from Yb:KGW laser (1030 nm, 220 fs Gaussian fit, 100 kHz, Light Conversion Ltd) was separated to two light beam. One was introduced to NOPA (ORPHEUS-N, Light Conversion Ltd) to produce a certain wavelength for pump beam (here we use 750 nm), the other was focused onto a YAG plate to generate white light continuum as probe beam. The pump and probe overlapped on the sample at a small angle less than 10°. The transmitted probe light from sample was collected by a linear CCD array. Then we obtained transient differential transmission signals by equation shown below:

$$\frac{\Delta T}{T} = \frac{T_{pump-on} - T_{pump-off}}{T_{pump-off}} \qquad (2)$$

Supplementary References

1. Gao, W. *et al.* Asymmetrical ladder-type donor-induced polar small molecule acceptor to promote fill factors approaching 77% for high-performance nonfullerene polymer solar cells. *Adv. Mater.* **30**, 1800052 (2018).
2. Li, C. *et al.* A nonfullerene acceptor utilizing a novel asymmetric multifused-ring core unit for highly efficient organic solar cells. *J. Mater. Chem. C* **6**, 4873-4877 (2018).
3. Song, J. *et al.* Extension of indacenodithiophene backbone conjugation enables efficient asymmetric A-D-A type non-fullerene acceptors. *J. Mater. Chem. A* **6**, 18847-18852 (2018).
4. Gao, W. *et al.* Designing an asymmetrical isomer to promote the LUMO energy level and molecular packing of a non-fullerene acceptor for polymer solar cells with 12.6% efficiency. *Chem. Sci.* **9**, 8142-8149 (2018).
5. Gao, W. *et al.* Asymmetrical small molecule acceptor enabling nonfullerene polymer solar cell with fill factor approaching 79%. *ACS Energy Lett.* **3**, 1760-1768 (2018).
6. Li, C. *et al.* High-performance eight-membered indacenodithiophene-based asymmetric A-D-A type non-fullerene acceptors. *Sol. RRL* **3**, 1800246 (2019).
7. Li, C. *et al.* Asymmetric selenophene-based non-fullerene acceptors for high-performance organic solar cells. *J. Mater. Chem. A* **7**, 1435-1441 (2019).
8. Li, X. *et al.* Asymmetric A-D-π-A-type nonfullerene small molecule acceptors for efficient organic solar cells. *J. Mater. Chem. A* **7**, 19348-19354 (2019).
9. Gao, W. *et al.* Dithieno[3,2-b:2′,3′-d]pyrrol-fused asymmetrical electron acceptors: A study into the effects of nitrogen-functionalization on reducing nonradiative recombination loss and dipole moment on morphology. *Adv. Sci.* **7**, 1902657 (2020).
10. Gao, W. *et al.* Simultaneously increasing open-circuit voltage and short-circuit current to minimize the energy loss in organic solar cells via designing asymmetrical non-fullerene acceptor. *J. Mater. Chem. A* **7**, 11053-11061 (2019).
11. Yang, L. *et al.* Tuning of the conformation of asymmetric nonfullerene acceptors for efficient organic solar cells. *J. Mater. Chem. A* **7**, 22279-22286 (2019).
12. Feng, S. *et al.* Fused-ring acceptors with asymmetric side chains for high-performance thick-film organic solar cells. *Adv. Mater.* **29**, 1703527 (2017).
13. Feng, S. *et al.* Controlling molecular packing and orientation via constructing a ladder-type electron acceptor with asymmetric substituents for thick-film nonfullerene solar cells. *ACS Appl. Mater. Interfaces* **11**, 3098-3106 (2019).
14. Ye, L. *et al.* Asymmetric fused-ring electron acceptor with two distinct terminal groups for efficient organic solar cells. *J. Mater. Chem. A* **7**, 8055-8060 (2019).
15. Aldrich, T. J. *et al.* Fluorination effects on indacenodithienothiophene acceptor packing and electronic structure, end-group redistribution, and solar cell photovoltaic response. *J. Am. Chem. Soc.* **141**, 3274-3287 (2019).
16. Li, M., Zhou, Y., Zhang, J., Song, J. & Bo, Z. Tuning the dipole moments of nonfullerene acceptors with an asymmetric terminal strategy for highly efficient organic solar cells. *J. Mater. Chem. A* **7**, 8889-8896 (2019).
17. Gao, B. *et al.* Multi-component non-fullerene acceptors with tunable bandgap structures for efficient organic solar cells. *J. Mater. Chem. A* **6**, 23644-23649 (2018).

18. Zhang, J. *et al.* One-pot synthesis of electron-acceptor composite enables efficient fullerene-free ternary organic solar cells. *J. Mater. Chem. A* **6**, 22519-22525 (2018).
19. Luo, Z. *et al.* Conformation-tuning effect of asymmetric small molecule acceptors on molecular packing, interaction, and photovoltaic performance. *Small* **16**, 2001942 (2020).
20. Luo, Z. *et al.* A pyrrole-fused asymmetrical electron acceptor for polymer solar cells with approaching 16% efficiency. *Small Struct.* **2**, 2000052 (2021).
21. Li, S. *et al.* Asymmetric electron acceptors for high-efficiency and low-energy-loss organic photovoltaics. *Adv. Mater.* **32**, 2001160 (2020).
22. Liu, T. *et al.* Asymmetric acceptors with fluorine and chlorine substitution for organic solar cells toward 16.83% efficiency. *Adv. Funct. Mater.* **30**, 2000456 (2020).
23. Gao, W. *et al.* Asymmetric acceptors enabling organic solar cells to achieve an over 17% efficiency: Conformation effects on regulating molecular properties and suppressing nonradiative energy loss. *Adv. Energy Mater.* **11**, 2003177 (2021).
24. Chen, H. *et al.* 17.1 %-efficient eco-compatible organic solar cells from a dissymmetric 3D network acceptor. *Angew. Chem. Int. Ed.* **60**, 3238-3246 (2021).
25. Luo, Z. *et al.* Fine-tuning energy levels via asymmetric end groups enables polymer solar cells with efficiencies over 17%. *Joule* **4**, 1236-1247 (2020).
