# Supplementary material for: Unveiling structure-performance relationships from multi-scales in non-fullerene organic photovoltaics
Source: Nat Commun. 2021 Jul 30;12:4627. doi: 10.1038/s41467-021-24937-5 (PMC8324909; doi:10.1038/s41467-021-24937-5)
Supplement: Supplementary file 3 — Solar Cells Reporting Summary [file 41467_2021_24937_MOESM3_ESM.pdf]

nature research

Corresponding author(s): Hongzheng Chen, Lijian Zuo

Solar Cells Reporting Summary

Nature Research wishes to improve the reproducibility of the work that we publish. This form is intended for publication with all accepted papers reporting the characterization of photovoltaic devices and provides structure for consistency and transparency in reporting. Some list items might not apply to an individual manuscript, but all fields must be completed for clarity.

For further information on Nature Research policies, including our data availability policy, see Authors & Referees.

Experimental design

Please check: are the following details reported in the manuscript?

1. Dimensions

Area of the tested solar cells

- [x] Yes — The area of the tested solar cells is 9.25 mm2, as illustrated in the Section of "Methods" in the Manuscript.
- [ ] No — *Explain why this information is not reported/not relevant.*

Method used to determine the device area

- [x] Yes — The device area is defined by the overlaping area of ITO and Ag grids, and this information is illustrated in Section of "Methods" in the manuscript
- [ ] No — *Explain why this information is not reported/not relevant.*

2. Current-voltage characterization

Current density-voltage (J-V) plots in both forward and backward direction

- [ ] Yes — *State where this information can be found in the text.*
- [x] No — Organic photovoltaic cells don't show the hysteresis (forward and backward scan).

Voltage scan conditions
For instance: scan direction, speed, dwell times

- [x] Yes — The scan direction is -0.2 V to 1.2 V, with a scan step of 0.01 V and dwell time is 1 ms, described in the section of "Methods" in the Manuscript.
- [ ] No — *Explain why this information is not reported/not relevant.*

Test environment
For instance: characterization temperature, in air or in glove box

- [x] Yes — Devices were tested in N2-filled glove box, and this information is illustrated in "Method" section in the revised manuscript.
- [ ] No — *Explain why this information is not reported/not relevant.*

Protocol for preconditioning of the device before its characterization

- [ ] Yes — *State where this information can be found in the text.*
- [x] No — No preconditioning protocol, because the performance of organic solar cell exhibit no preconditioning dependence.

Stability of the J-V characteristic
Verified with time evolution of the maximum power point or with the photocurrent at maximum power point; see ref. 7 for details.

- [ ] Yes — *State where this information can be found in the text.*
- [x] No — Not tested, because the J-V stability issue is not a key claim in OPV as we understand.

3. Hysteresis or any other unusual behaviour

Description of the unusual behaviour observed during the characterization

- [ ] Yes — *State where this information can be found in the text.*
- [x] No — Generally, there is no hysteresis in the organic photovoltaic cells. No unusual behaviours, such as hysteresis, were observed in our devices during the measurement.

Related experimental data

- [ ] Yes — *State where this information can be found in the text.*
- [x] No — No unusual behaviour was found.

4. Efficiency

External quantum efficiency (EQE) or incident photons to current efficiency (IPCE)

- [x] Yes — EQE is provided as Fig. 2b in the manuscript.
- [ ] No — *Explain why this information is not reported/not relevant.*

A comparison between the integrated response under the standard reference spectrum and the response measure under the simulator

- [x] Yes — The integrated JSC values calculated from the EQE curves are consistent with those obtained from the J-V curves (within 1.5% error).
- [ ] No — *Explain why this information is not reported/not relevant.*

For tandem solar cells, the bias illumination and bias voltage used for each subcell

- [ ] Yes — *State where this information can be found in the text.*
- [x] No — No tandem solar cells are fabricated in our work.

5. Calibration

Item	Yes/No	Response
Light source and reference cell or sensor used for the characterization	☒ Yes ☐ No	The light source is a Class 3A solar simulator (SS-F5-3A, Enlitech). The light intensity is calibrated with a 20 mm×20 mm monocrystalline silicon reference cell with KG2 filter (purchased from Enli Tech. Co., Ltd., Taiwan, which has been Calibrated by authority party). The details can be found in the Section of "Methods" in the manuscript. *Explain why this information is not reported/not relevant.*
Confirmation that the reference cell was calibrated and certified	☒ Yes ☐ No	The standard monocrystalline silicon reference cell with KG2 filter was purchased from Enli Tech. Co., Ltd., Taiwan and was calibrated and certified. The details can be found in the Section of "Methods" in the manuscript. *Explain why this information is not reported/not relevant.*
Calculation of spectral mismatch between the reference cell and the devices under test	☐ Yes ☒ No	*State where this information can be found in the text.* We did not calculate the mismatch between the reference cell and the devices under test, for this value is very small with 3A solar simulator after calibration reference cell with KG-2 filter, and this is also confirmed with the certified device performance from a third authorized party.

6. Mask/aperture

Item	Yes/No	Response
Size of the mask/aperture used during testing	☒ Yes ☐ No	A mask with an area of 5.979 mm2 was used during testing, as illustrated in "Method" section. *Explain why this information is not reported/not relevant.*
Variation of the measured short-circuit current density with the mask/aperture area	☒ Yes ☐ No	See the Jsc statistics in Table 1. *Explain why this information is not reported/not relevant.*

7. Performance certification

Item	Yes/No	Response
Identity of the independent certification laboratory that confirmed the photovoltaic performance	☒ Yes ☐ No	The best-performing PM6:BTP-S9-based OPVs were sent to National Institute of Metrology (NIM), China for certification, as illustrated in "Method" section, and a certified efficiency of 17.4% was achieved, as shown in Supplementary Fig. 6. *Explain why this information is not reported/not relevant.*
A copy of any certificate(s) *Provide in Supplementary Information*	☒ Yes ☐ No	The copy of efficiency certification report is provided as Supplementary Fig. 6. *Explain why this information is not reported/not relevant.*

8. Statistics

Item	Yes/No	Response
Number of solar cells tested	☒ Yes ☐ No	At least 10 independent devices were tested for the calculation of average device parameters, as described in Table 1. *Explain why this information is not reported/not relevant.*
Statistical analysis of the device performance	☒ Yes ☐ No	Statistical analysis of the device performances are provided in Table 1. *Explain why this information is not reported/not relevant.*

9. Long-term stability analysis

Item	Yes/No	Response
Type of analysis, bias conditions and environmental conditions *For instance: illumination type, temperature, atmosphere humidity, encapsulation method, preconditioning temperature*	☒ Yes ☐ No	Irradiance stability under AM 1.5 G illumination in N2 atmosphere was tested for unencapsulated devices. Thermal stability at 80 oC in N2 atmosphere was tested for unencapsulated devices. The results are provided in Fig. 2c and Supplementary Fig. 9. *Explain why this information is not reported/not relevant.*
